# Exploring the role of innate immunity in Cholangiocarcinoma: implications for prognosis, immune infiltration, and tumor metastasis

**DOI:** 10.7150/jca.94194

**Published:** 2024-05-05

**Authors:** Wenhuan Sun, Jianrong Ge, Long Zhang, Fangfang Zhou, Jisheng Liu

**Affiliations:** 1 The first Affiliated Hospital of Soochow University, Institutes of Biology and Medical Sciences of Soochow University, Suzhou Medical College of Soochow University, Suzhou, China.; 2 Department of Otolaryngology Head and Neck Surgery, The First Affiliated Hospital of Soochow University, Suzhou, China.; 3 MOE Laboratory of Biosystems Homeostasis & Protection and Innovation Center for Cell Signaling Network, Life Sciences Institute, Zhejiang University, Hangzhou, China Cancer Center, Zhejiang University, Hangzhou, China.

**Keywords:** innate immune pathway, cholangiocarcinoma, prognosis, immune cell infiltration, metastasis

## Abstract

The innate immune system serves as the body's primary physiological defense against the intrusion of pathogenic microorganisms, playing a pivotal role in restricting viral infections. However, current research on the interplay between innate immune pathways and cancer is limited, with reported effects often inconsistent. Therefore, we aimed to elucidate the relationship between innate immune pathways and tumors through an amalgamation of bioinformatics and extensive data analysis. Conducting a pan-cancer analysis encompassing expression, genomic alterations, and clinical prognosis, we identified a close association between the innate immune pathway and cholangiocarcinoma. Subsequently, our focus shifted to unraveling the role of innate immune pathway proteins in cholangiocarcinoma. TIMER database analysis showed that the innate immune pathway predominantly influences the infiltration of macrophages and B cells in cholangiocarcinoma. Additionally, gene ontology (GO) and pathway analyses were performed for significantly differentially expressed genes correlated with the innate immune pathway in cholangiocarcinoma. Single-cell transcriptome analysis in cholangiocarcinoma demonstrated that genes in the innate immune pathway are primarily expressed in malignant cells, endothelial cells, monocytes and macrophages. To further validate the expression of proteins in the innate immune pathway in the tumor tissues of patients with cholangiocarcinoma, tumor tissue slices from patients with liver intrahepatic cholangiocarcinoma and normal tissue slices from the HPA database were analyzed. These results indicated pronounced activation of the innate immune pathway in the tumor tissues of patients with cholangiocarcinoma. Finally, proteomic data from patients with or without intrahepatic cholangiocarcinoma metastasis were analyzed. The results revealed a significant correlation between the expression and phosphorylation of IKKε and the occurrence of intrahepatic cholangiocarcinoma metastasis. These findings not only demonstrate the significance of the innate immune pathway in cholangiocarcinoma but also its potential as a prospective prognostic biomarker and therapeutic target for this malignancy.

## Introduction

The innate immune system provides an expeditious defense mechanism through the identification of molecular structures emanating from microbial pathogens. This facilitates the orchestration of adaptive immune responses to antigen-specific counteraction. Cellular pattern recognition receptors (PRRs) are activated by the recognition of viral pathogen-associated molecular patterns (PAMPs). These include retinoic acid-inducible gene I (RIG-I)-like receptors, Toll-like receptors, cytosolic DNA sensors, and nucleotide-binding oligomerization domain-like receptors 1-4. Activated PRRs subsequently recruit the adaptor proteins MAVS or STING, thereby instigating TBK1/IKKε kinase cascades, culminating in the induction of interferon type I (IFN-I) and proinflammatory cytokines 5, 6.

In addition to its role as the primary defense mechanism of organisms against external pathogens such as viruses, the innate immune response plays a pivotal role in anti-tumor immunity. Chromosomal instability (CIN) is a hallmark feature of tumors that stems from the persistent erroneous segregation of chromosomes during the late stages of mitosis 7, 8. This instability propels the evolutionary trajectory of neoplastic transformation, leading to the generation of misplaced DNA in the form of micronuclei and chromatin bridges in cancer cells. Subsequently, these aberrations are recognized by the nucleic acid sensor cGAS, which activates the downstream signaling protein STING. The activation of this immune pathway stimulates the mobilization and activation of immune cells, resulting in the elimination of cancer cells 9. Nevertheless, evidence suggests that chronic activation of the cGAS-STING pathway may contribute to inflammation-driven carcinogenesis 10-12. Additionally, RNA accumulation in tumor cells is recognized by RIG-I, which activates the RIG-I-MAVS signaling cascade, thereby recruiting immune cells to the tumor microenvironment 13. Furthermore, studies have substantiated the utilization of RIG-I agonists, such as 5'-pppRNA, for tumor treatment 14-16. However, as a downstream adaptor protein of RIG-I, recent studies have demonstrated that MAVS influences dendritic cells (DCs) by inhibiting the production of IL-12, thereby impairing the functional efficacy of CD8+ T cells and facilitating tumor cell escape 17.

In summary, current research on the interplay between innate immunity and tumors has predominantly focused on the inhibitory and stimulatory roles of the innate immune pathway in oncogenesis. However, the existing literature on the relationship between innate immune pathways and tumors currently lacks consensus, and investigations into the connections between other pivotal proteins within the innate immune pathways and the initiation and progression of tumors remain insufficient. Hence, we aimed to employ bioinformatics methodologies and harness the power of multi-omics big data to comprehensively scrutinize the intricate connections between innate immune pathways and tumorigenesis. This study aimed to identify novel prognostic and therapeutic targets for future advancements in cancer prevention and treatment.

## Materials and Methods

### Gene expression and analysis

We entered the key genes of innate immune pathway (*DDX58*, *MAVS*, *C6orf150*, *TMEM173*, *IKBKE*, *TBK1* and *IRF3*) into the Gene Expression Analysis module of the TIMER2 website (http://timer.cistrome.org/) 18. Subsequently, we analyzed the expression patterns of these genes across various tumor and normal tissues. For validation purposes, we utilized another gene analysis database, GEPIA2 (http://gepia2.cancer-pku.cn/#analysis) 19.

Immunohistochemical information for intrahepatic cholangiocarcinoma and normal tissue slices was downloaded from the Human Protein Atlas (HPA; https://www.proteinatlas.org/). This data was employed to analyze the expression levels of proteins in different samples.

### Genetic alteration analysis

cBioPortal (www.cbioportal.org) is a comprehensive online resource offering visual and multidimensional data on cancer genomics 20. To assess the genetic variation characteristics of the *DDX58*, *MAVS*, *C6orf150*, *TMEM173*, *IKBKE*, *TBK1,* and *IRF3* genes, we utilized cBioPortal. Specifically, we selected the “TCGA PanCancer Atlas Studies-Query By Gene” in the “Quick select” section and entered “*DDX58*, *MAVS*, *C6orf150*, *TMEM173*, *IKBKE*, *TBK1* and *IRF3*.” The alteration frequency, mutation type, and Copy Number Alteration (CNA) of these genes in datasets such as Breast Invasive Carcinoma (TCGA, PanCancer Atlas), Lung Squamous Cell Carcinoma (TCGA, PanCancer Atlas), Liver Hepatocellular Carcinoma (TCGA, PanCancer Atlas), Cholangiocarcinoma (TCGA, Firehose Legacy), and Colorectal Adenocarcinoma (MSK, Nat Commun 2022) were observed in the “Cancer Types Summary” module. The mutated sites of *DDX58*, *MAVS*, *C6orf150*, *TMEM173*, *IKBKE*, *TBK1* and *IRF3* were illustrated in schematic diagrams of the protein structure or the three-dimensional (3D) structure through the “Mutations” module.

### Survival prognosis analysis

We used the GEPIA2 database to analyze the Overall Survival (OS) and disease-free survival (DFS) data related to innate immune pathway proteins in various tumors. High cutoff (50%) and low cutoff (50%) values were employed as expression thresholds to delineate the high- and low-expression cohorts. The log-rank test was used for the hypothesis test, and the Kaplan-Meier Survival Analysis module of GEPIA2 was utilized to generate the survival curve.

### LinkedOmics analysis

We utilized the TCGA_CHOL dataset (HiSeq RNA platform) in LinkedOmics to analyze the genes associated with innate immune pathway proteins. Gene set enrichment analysis (GSEA) was employed for KEGG pathway analysis. Additionally, gene classification was conducted using gene ontology (GO) to categorize genes based on their biological processes, cellular components, and molecular functions.

### Immune infiltration analysis

We utilized the TIMER database to scrutinize the infiltration of six distinct immune cell types in cholangiocarcinoma, including B cells, CD4+ T cells, CD8+ T cells, neutrophils, macrophages, and dendritic cells 21. Utilizing the correlation analysis module within this database, we evaluated the correlation between the expression of target genes and the infiltration of immune cells.

### Promoter methylation analysis

The UALCAN portal (https://ualcan.path.uab.edu/index.html) was used to analyze the differences in promoter methylation levels of innate immune pathway proteins between cholangiocarcinoma patients and normal controls.

### TISCH2 database analysis

TISCH2 offers detailed annotations of immune cell types at the single-cell level, aiding users in exploring the tumor microenvironment in various cancers 22. We examined single-cell transcriptomic datasets GSE138709 and GSE142784 from the TISCH2 database to assess the expression profiles of pivotal proteins within the innate immune pathway across diverse cellular subtypes of cholangiocarcinoma.

## Results

### Pan-cancer analysis of innate immune pathway

**Figure 1:**
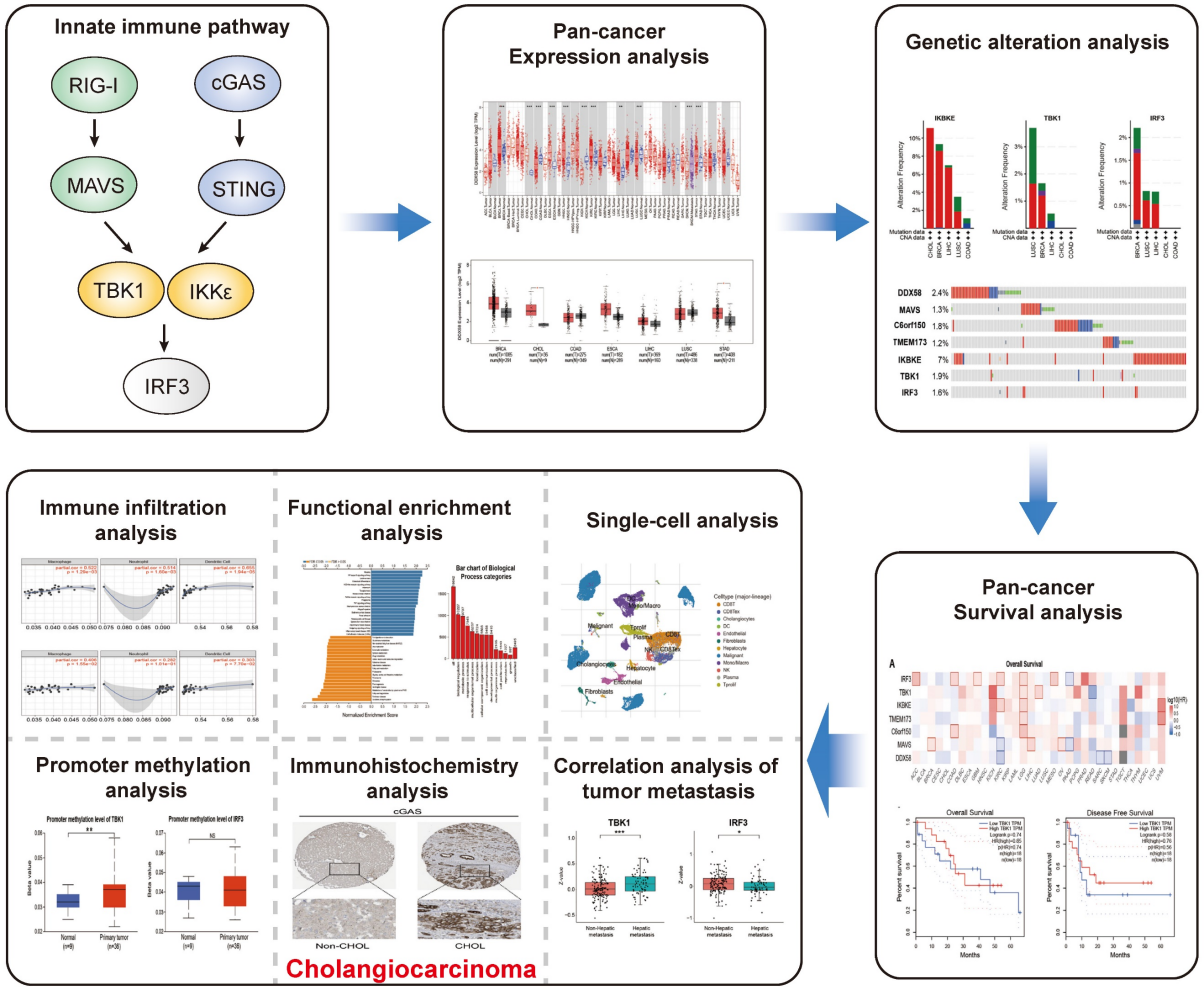
The schematic workflow representing the study's design and the data collection. Primarily, a comprehensive analysis of the pan-cancer expression, genomic alterations and survival prognosis of key proteins within the innate immune pathway. Subsequently, functional enrichment analysis of relevant genes, immune infiltration analysis, single-cell transcriptomic analysis, immunohistochemical analysis, promoter methylation analysis and correlation analysis with tumor metastasis, all focused on the key proteins within the innate immune pathway in cholangiocarcinoma.

**Figure 2:**
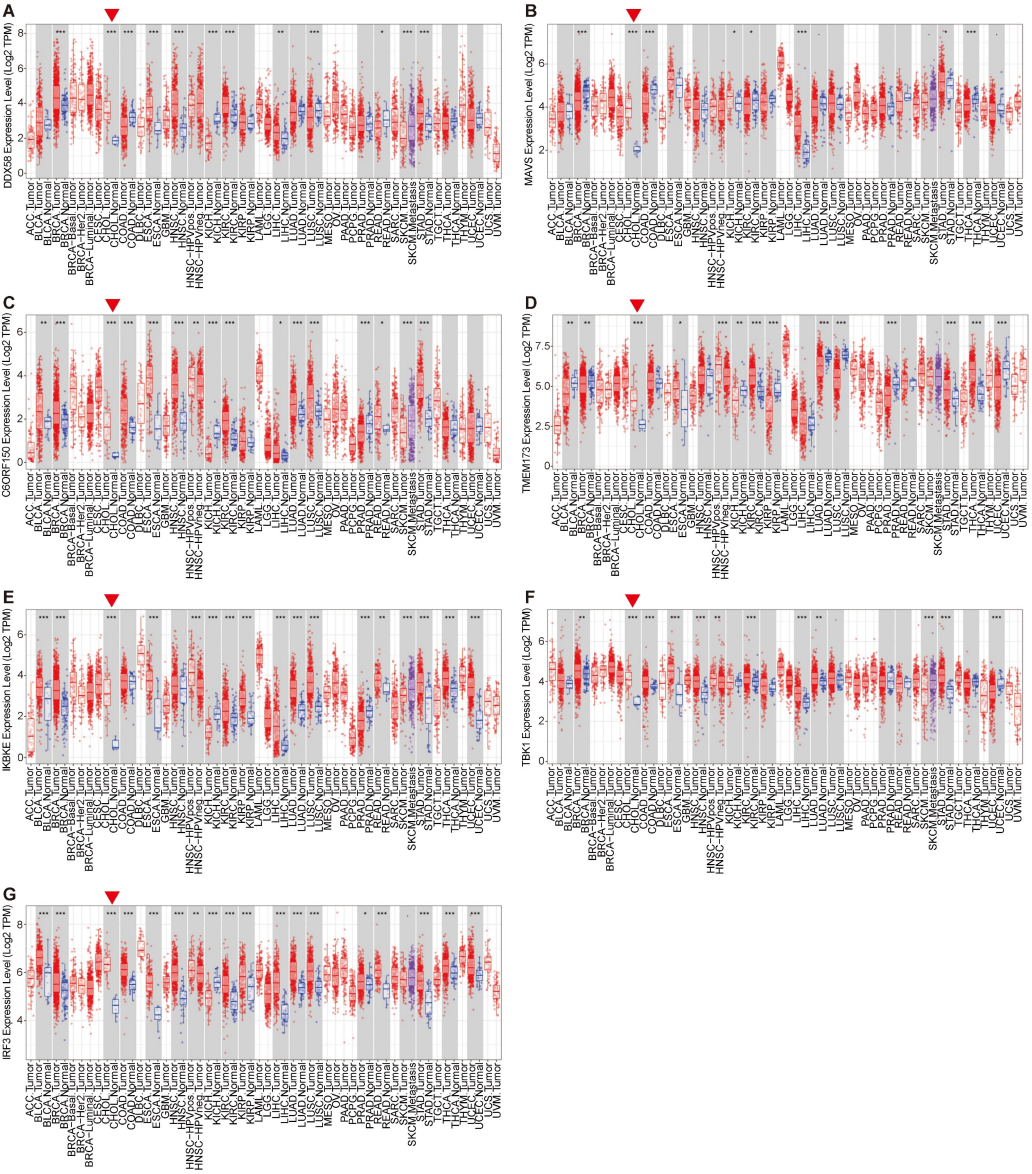
Innate immune pathway expression levels in different types of human cancers. Key genes of innate immune pathway expression levels in different tumor types from TCGA were determined by TIMER (**A-G**) (*P < 0.05, **P < 0.01, ***P < 0.001).

An illustrative schematic delineating the conceptual framework and methodology for data acquisition is shown in Figure 1. Initially, we examined the expression levels of key genes within the innate immune pathway, namely *DDX58*, *MAVS*, *C6orf150*, *TMEM173*, *IKBKE*, *TBK1* and *IRF3*, across diverse tumor and non-tumor tissues. The TIMER2 database facilitated the analysis of *DDX58*, *MAVS*, *C6orf150*, *TMEM173*, *IKBKE*, *TBK1,* and *IRF3* expression in various TCGA tumors. Remarkably, our findings revealed a consistent upregulation in the expression levels of these genes in CHOL (cholangiocarcinoma). Specifically, *DDX58*, *C6orf150*, *IKBKE*, *TBK1,* and *IRF3* exhibited a pronounced increase in breast invasive carcinoma (BRCA), while *MAVS* and *TMEM173* expression was significantly lower in BRCA compared to normal controls (Figure 2A-G).

By utilizing the GEPIA2 database in tandem with the TIMER2 database, we further evaluated the expression of *DDX58*, *MAVS*, *C6orf150*, *TMEM173*, *IKBKE*, *TBK1* and *IRF3* in BRCA, CHOL, colon adenocarcinoma (COAD), esophageal carcinoma (ESCA), liver hepatocellular carcinoma (LIHC), lung squamous cell carcinoma (LUSC) and stomach adenocarcinoma (STAD) in normal and tumor tissues. These results are consistent with the results of TIMER2 database analysis, where the expression levels of *DDX58*, *MAVS*, *C6orf150*, *TMEM173*, *IKBKE*, *TBK1* and *IRF3* were all higher than those in the corresponding normal tissues (Figure S1A-G).

**Figure 3:**
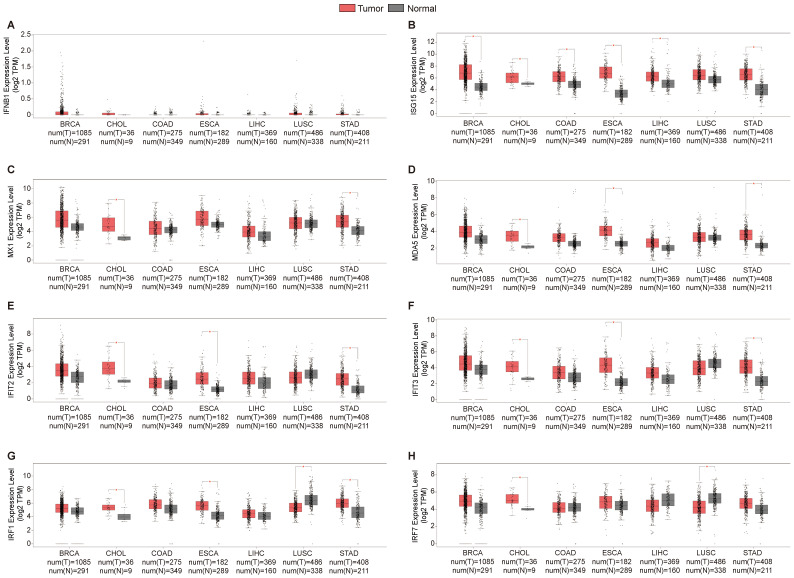
Expression levels of the Type I interferons and interferon-stimulated genes (ISG). For the types of BRCA, CHOL, COAD, ESCA, LIHC, LUSC and STAD in the TCGA project, the corresponding normal tissues in the GTEx database were included as controls. The box plot data were supplied. (*P<0.01).

The activation of the innate immune pathway involves not only the autonomous action of pathway proteins but also induces the production of interferons, subsequently prompting the expression of downstream interferon-stimulated genes (ISGs), initiating an immune response. Therefore, we conducted a more in-depth analysis of the roles of Type I interferons and their downstream ISG genes in tumors. We analyzed the expression levels of Type I interferons and ISG genes in the tumor tissues of patients and normal control tissues, revealing a consistent upregulation of ISG genes, including *ISG15*, *MX1*, *MDA5*,* IFIT2*,* IFIT3*, *IRF1* and *IRF7*, in the tumor tissues of patients with cholangiocarcinoma. However, the expression of the type I interferon *IFNB1* remained insignificantly altered across multiple tissues (Figure 3A-H). Overall, these results are consistent with earlier findings on innate immune pathway proteins in cholangiocarcinoma. This may be attributed to the high expression of innate immune pathway proteins in cholangiocarcinoma, which consequently induces ISG expression.

### Genetic alteration analysis of innate immune pathway in cancer

**Figure 4:**
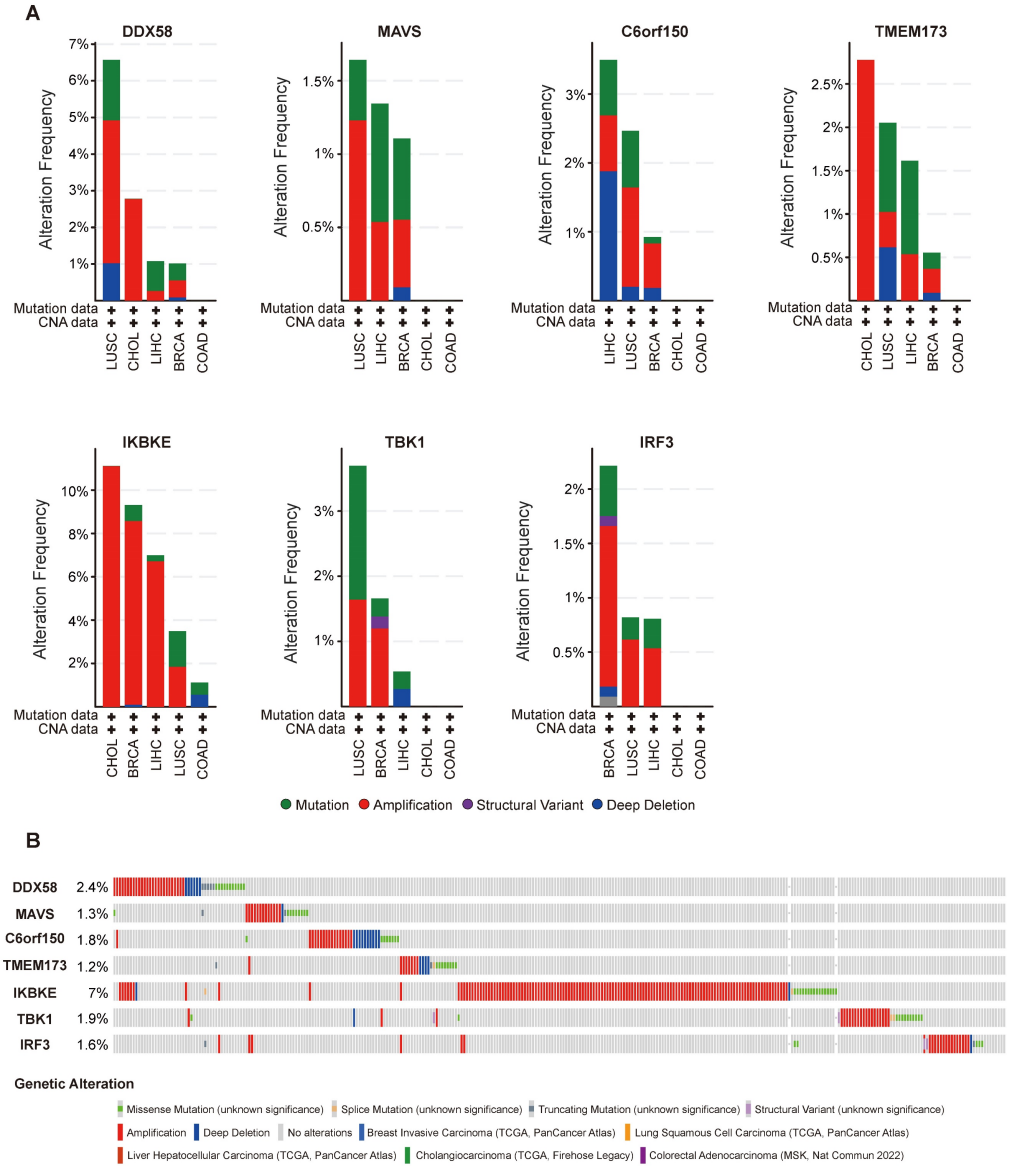
Innate immune pathway alternation analysis in different tumors. (**A**) The proportions of various innate immune pathway alternations in different subclasses of tumors. (**B**) The genetic alternations of innate immune pathway in tumors.

Alterations in the expression of *DDX58*, *MAVS*, *C6orf150*, *TMEM173*, *IKBKE*, *TBK1* and *IRF3* were analyzed across different tumor samples using cBioPortal. The examined alteration types included mutations, fusions, amplifications, deep deletions, and various combinations. Interestingly, key proteins within the innate immune pathway exhibited notable alterations across various tumor subtypes. Particularly, noteworthy alteration frequencies were observed for *DDX58* (~4%) and *MAVS* (~1.2%) in patients with LUSC, predominantly characterized by “Amplification.” In LIHC, the predominant type of copy number alteration (CNA) identified was “deep deletion,” with an alteration frequency of *C6orf150* (~2%). In addition, the highest alteration frequencies for *TMEM173* (~2.5%) and *IKBKE* (~10%) were observed in patients with CHOL, primarily characterized by “amplification”. The highest alteration frequency of *IRF3* (~1.5%) was observed in patients with BRCA, which was predominantly marked by “amplification”. LUSC exhibited the principal type of CNA as “Mutation,” with an alteration frequency of *TBK1* (~1.5%) (Figure 4A). In addition, *DDX58*, *MAVS*, *C6orf150*, *TMEM173*, *IKBKE*, *TBK1,* and *IRF3* showed alterations in 2.4%, 1.3%, 1.8%, 1.2%, 7%, 1.9%, and 1.6% of multiple tumor samples, respectively (Figure 4B).

**Figure 5:**
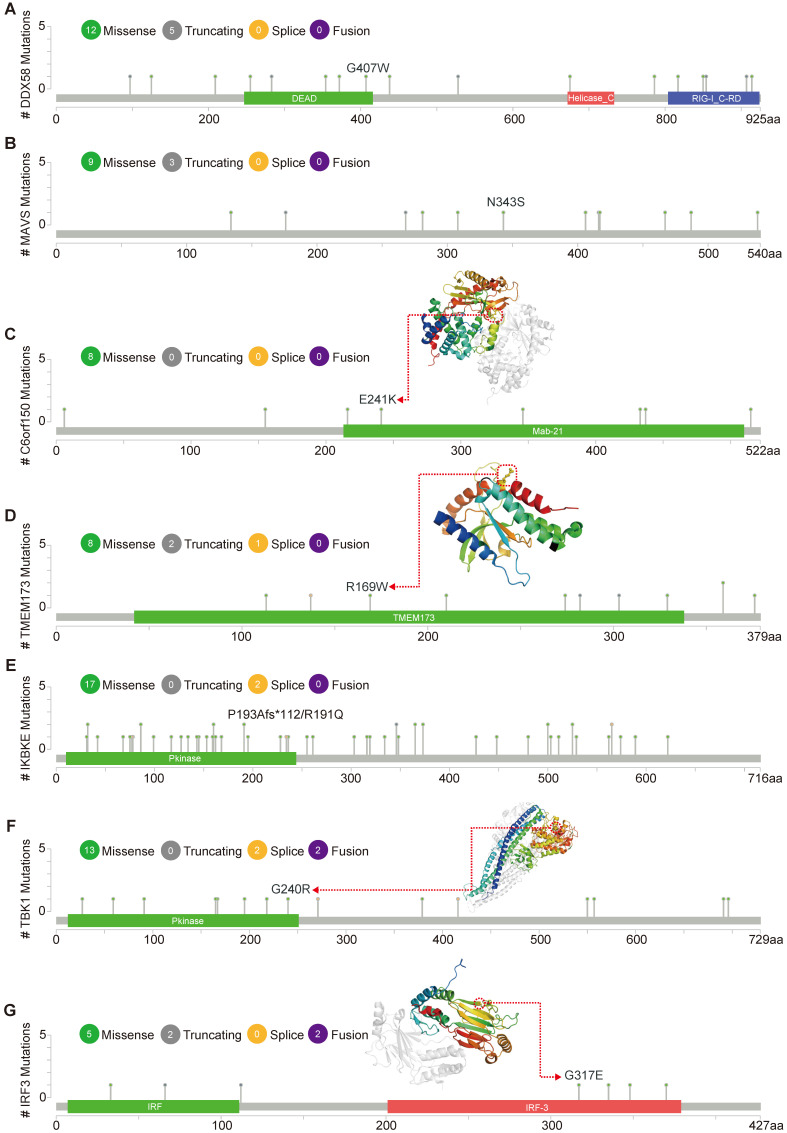
Mutation features of innate immune pathway in different tumors of TCGA. Mutation sites and the mutation site in the 3D structure of proteins are displayed (**A-G**).

Figure 5 illustrates the types, genomic loci, and frequencies of genetic alterations in *DDX58*, *MAVS*, *C6orf150*, *TMEM173*, *IKBKE*,* TBK1*, and* IRF3*. Missense mutations in *DDX58*, *MAVS*, *C6orf150*, *TMEM173*, *IKBKE*, *TBK1* and *IRF3* were identified as the main types of genetic alterations. Mutations in *DDX58*, *C6orf150*, *TMEM173*, and *TBK1* have been identified in LUSC. The genome pattern diagram depicts the 3D structures of cGAS (encoding *C6orf150*), STING (encoding *TMEM173*), TBK1, and IRF3.

### Prognostic potential of innate immune pathway in tumors

To ascertain the association between the expression of key proteins in innate immune pathways and clinical characteristics, we utilized TCGA and GEO datasets. The aim was to explore the role of innate immune pathways in tumors and their correlation with the prognosis of diverse cancer patients.

**Figure 6:**
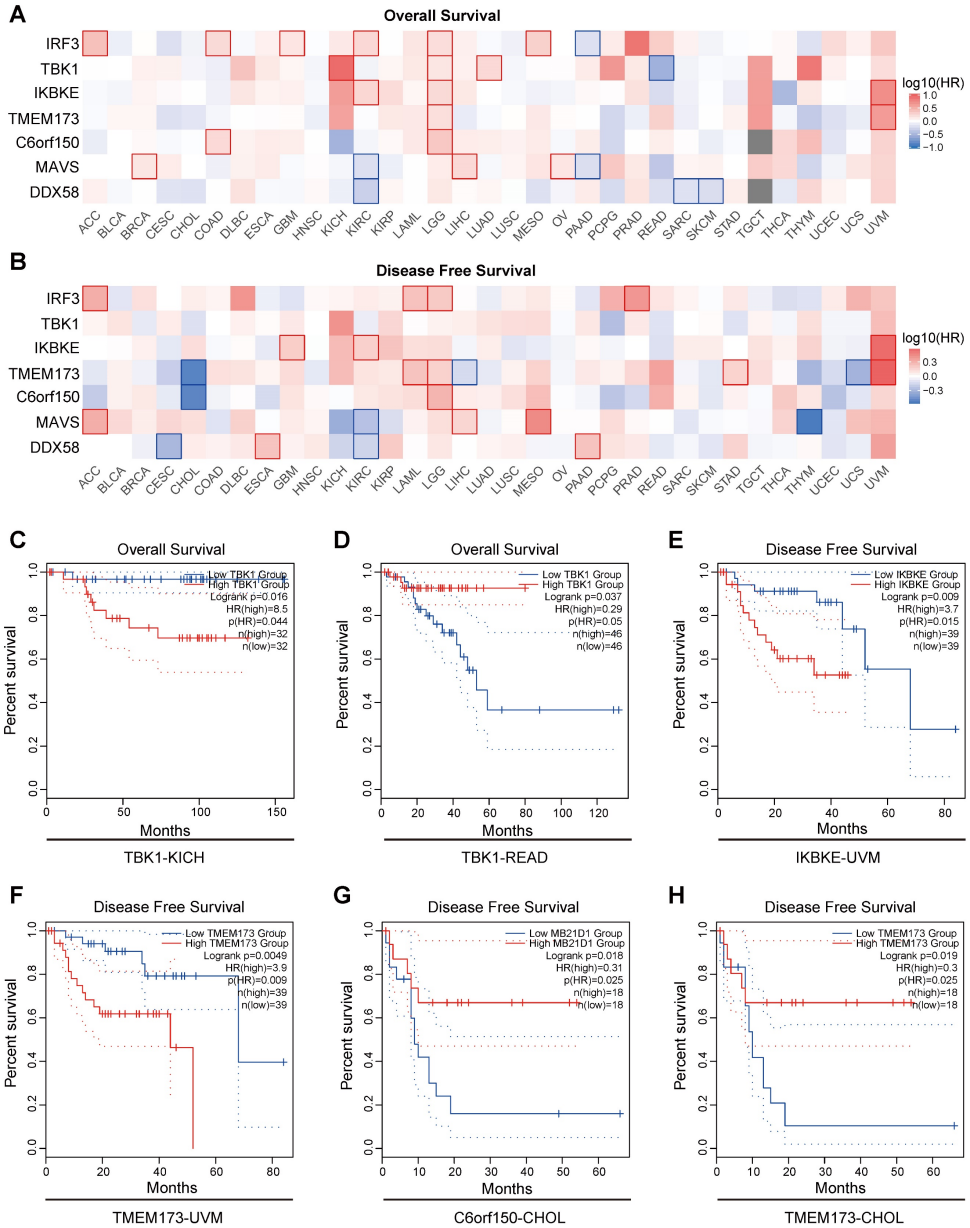
Prognostic significance of innate immune pathway in various cancer patients. Heatmap showing the impact of innate immune pathway on OS (**A**) and DFS (**B**). The survival curves for OS of *TBK1* in KICH patients (**C**), OS of *TBK1* in READ patients (**D**), DFS of *IKBKE* in UVM patients (**E**), DFS of *TMEM173* in UVM patients (**F**), DFS of *C6orf150* in CHOL patients (**G**), and DFS of *TMEM173* in CHOL patients (**H**) in TCGA data.

The results indicate a close association between the expression of proteins in the innate immune pathways and the survival outcomes of numerous cancer patients. Elevated expression of innate immune pathway proteins is associated with poorer OS (Overall Survival) in patients with KICH (Kidney Chromophobe), LGG (brain lower-grade glioma), and UVM (Uveal Melanoma) tumors. Moreover, adrenocortical carcinoma (ACC), Acute Myeloid Leukemia (LAML), LGG, mesothelioma (MESO), prostate adenocarcinoma (PRAD), and UVM tumors were linked to inferior disease-free survival (DFS) (Figure 6A, B). Here, we present several noteworthy findings that significantly influence OS and DFS. The result reveal that elevated expression of *TBK1* is associated with poorer OS in patients with KICH (*p* =0.016) (Figure 6C). However, in patients with READ, elevated expression of *TBK1* is associated with better OS (*p* =0.037) (Figure 6D). High expression levels of *IKBKE* and *TMEM173* were associated with poorer DFS in patients with UVM (*p* =0.009 and *p* =0.0049) (Figure 6E, F). Conversely, elevated expression of *C6orf150* and *TMEM173* in CHOL patients indicated a more favorable DFS (*p* =0.018 and *p* =0.019, respectively) (Figure 6G, H).

Considering the expression levels of key proteins in innate immune pathways, genomic alterations, and their correlation with clinical prognosis across multiple tumors, we observed a consistent and significant impact of innate immune pathways in cholangiocarcinoma. Consequently, our subsequent analysis will primarily focus on cholangiocarcinoma, delving into the specific role of innate immune pathways in this context.

### Innate immune pathway is closely correlated with immune infiltration

It has been posited that the “tumor microenvironment transcends the role of a passive observer, assuming the role of an active instigator in propelling cancer progression” 23. Tumor-infiltrating immune entities, including T cells, B cells, neutrophils, cancer-associated fibroblasts, tumor-associated macrophages, dendritic cells, myeloid-derived suppressor cells, and endothelial cells, are pivotal constituents of the intricate tapestry of the tumor microenvironment 24-26.

**Figure 7:**
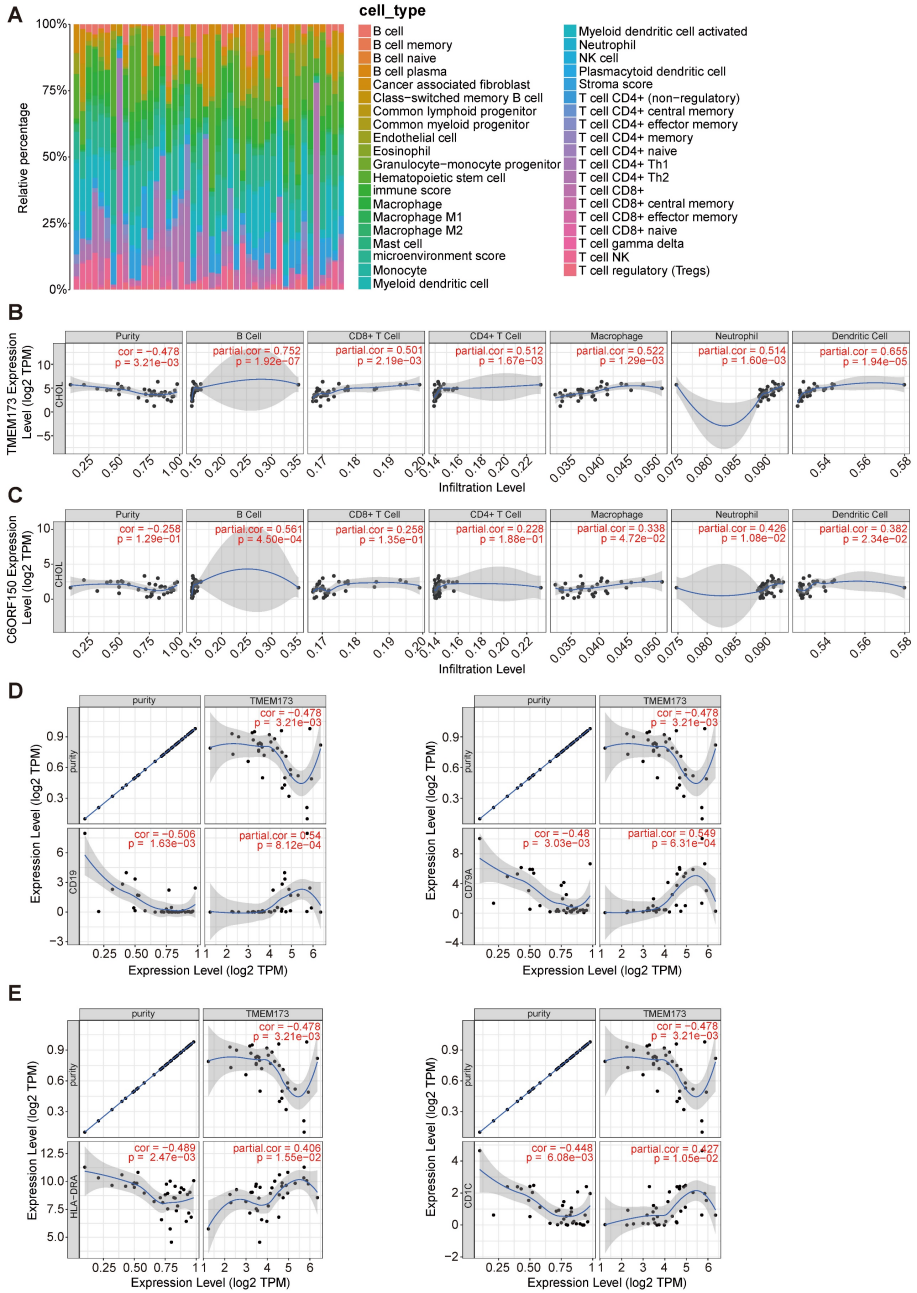
The correlation between differentially expressed innate immune pathway and immune cell infiltration in cholangiocarcinoma. The stacked histogram showing the composition of immune cells infiltrating in cholangiocarcinoma tissues (**A**). The correlations between the transcriptional levels of *TMEM173* (**B**) and *C6orf150* (**C**) with the infiltration of B cells, CD8+ T cells, CD4+ T cells, macrophages, neutrophils, and dendritic cells in cholangiocarcinoma were shown. Scatterplots of correlations between *TMEM173* expression and gene markers of B cell (**D**) or dendritic cell (**E**).

Initially, we employed the “xCell” methodology to analyze the transcriptome data of cholangiocarcinoma in the TCGA database, revealing a substantial proportion of macrophages, monocytes, and T cells within the tissue (Figure 7A). The TIMER database was then employed to examine the association between the expression levels of *DDX58*, *MAVS*, *C6orf150*, *TMEM173*, *IKBKE*, *TBK1*, and *IRF3* and the infiltration of six distinct types of immune cells (B cells, CD8+ T cells, CD4+ T cells, macrophages, neutrophils, and dendritic cells) in cholangiocarcinoma.

Tumor purity is a crucial factor in immune infiltration analysis. Our findings indicated that *DDX58*, *MAVS*, *C6orf150*, *IKBKE*, *TBK1,* and *IRF3* expression were not significantly correlated with tumor purity (Figure 7C and Figure S2). In contrast, *TMEM173* expression demonstrated a correlation with tumor purity (r = -0.478, *p* = 3.21e-3) (Figure 7B). The expression levels of *DDX58* positively correlated with the levels of infiltrating macrophages (r = 0.45, *p* = 6.69e-3) and neutrophils (r = 0.576, *p* = 2.92e-4) in CHOL (Figure S2). Expression levels of *C6orf150* were positively correlated with the levels of infiltrating B cells (r = 0.561, *p* = 4.50e-4), macrophages (r = 0.338, *p* = 4.72e-2), neutrophils (r = 0.426, *p* = 1.08e-2), and DCs (r = 0.382, *p* = 2.34e-2) in CHOL (Figure 7C). Expression levels of *TMEM173* were positively correlated with the levels of infiltrating B cells (r = 0.752, *p* = 1.92e-7), CD8+ T cells (r = 0.501, *p* = 2.19e-3), CD4+ T cells (r = 0.512, *p* = 1.67e-3), macrophages (r = 0.522, *p* = 1.29e-3), neutrophils (r = 0.514, *p* = 1.60e-3) and DCs (r = 0.655, *p* = 1.94e-5) in CHOL (Figure 7B). Expression levels of *IKBKE* were positively correlated with the number of infiltrating B cells (r = 0.398, *p* = 1.78e-2), CD4+ T cells (r = 0.399, *p* = 1.75e-2), and macrophages (r = 0.406, *p* = 1.55e-2) in CHOL. The expression levels of *TBK1* positively correlated with the levels of infiltrating B cells (r = 0.399, *p* = 1.76e-2), CD4+ T cells (r = 0.358, *p* = 3.46e-2), and neutrophils (r = 0.496, *p* = 2.47e-3) in CHOL (Figure S2). Furthermore, we conducted an additional analysis to examine the correlation between *TMEM173* and the B cell marker genes *CD19* and *CD79A*, as well as its correlation with the dendritic cell marker genes *HLA-DRA* and *CD1C*. The results indicated a significant correlation between the expression of *TMEM173* and the marker genes of both B cells and dendritic cells, suggesting that the expression of *TMEM173* plays a crucial role in regulating the function of B cells and DCs, exerting a significant influence on immune infiltration (Figure 7D, E). These findings strongly suggest that key proteins of the innate immune pathway play an important role in immune cell infiltration in the CHOL microenvironment, especially in the infiltration of B cells, CD8+ T cells, and macrophages.

### Analysis of Differentially Expressed Genes (DEGs) correlated with innate immune pathway in cholangiocarcinoma

**Figure 8:**
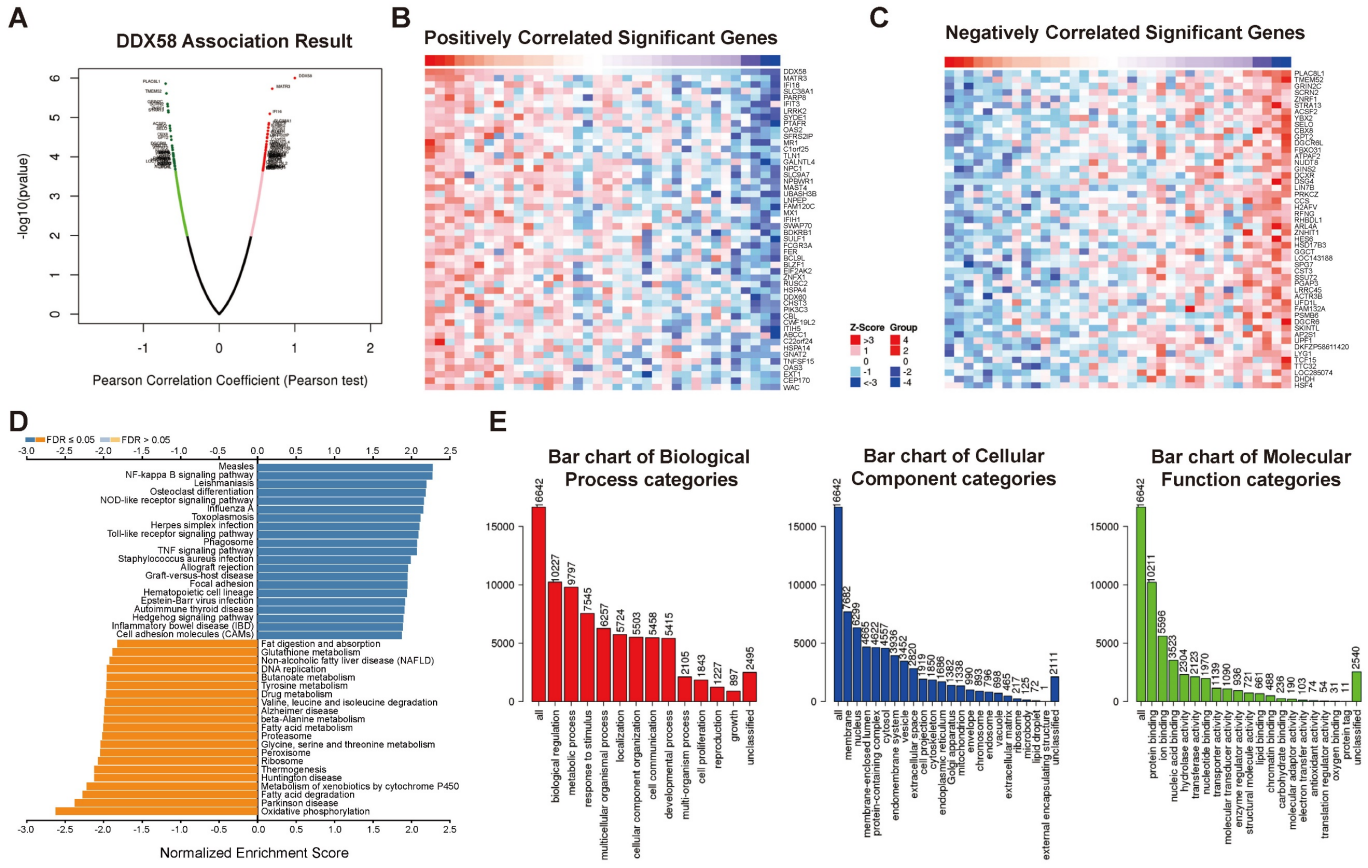
Analysis of differentially expressed genes in correlation with *DDX58* in cholangiocarcinoma. (**A**) Volcano plot showing the up-regulated and down-regulated genes correlated with *DDX58* expression (Pearson test). The significantly positively correlated (**B**) and negatively correlated (**C**) genes were shown in heatmaps. (**D**) Kyoto Encyclopedia Genes and Genomes (KEGG) pathway analysis of the significantly differentially expressed genes in correlation with *DDX58*. (**E**) Gene ontology (GO) analysis of the significantly differentially expressed genes in correlation with *DDX58*.

The DEGs associated with *DDX58* in cholangiocarcinoma were analyzed by LinkedOmics, presenting the results in the form of a volcano plot (Figure 8A). The top 50 genes demonstrating the most positive and negative correlations with *DDX58* were identified and depicted through heat maps (Figure 8B, C). Subsequently, significant DEGs underwent KEGG pathway enrichment analysis using Gene Set Enrichment Analysis (GSEA). Notably, the NF-kappa B signaling pathway exhibited significant upregulation, while oxidative phosphorylation, fatty acid degradation, and metabolism of xenobiotics by cytochrome P450 were markedly downregulated in cholangiocarcinoma (Figure 8D). Conducting Gene Ontology (GO) enrichment analysis, we identified the three most enriched biological process terms as biological regulation, metabolic processes, and response to stimulus. Additionally, the three most enriched cellular component terms were membrane, nucleus, and membrane-enclosed lumen, while the three most enriched molecular function terms were protein-, ion-, and nucleic acid-binding (Figure 8E). Supplementary Figures S3-8 display the classification and enrichment results for DEGs correlated with *MAVS*, *C6orf150*, *TMEM173*, *IKBKE*, *TBK1,* and *IRF3*. GSEA further indicated that* MAVS*, *C6orf150*, *TMEM173*, *IKBKE*, *TBK1,* and* IRF3* genes were correlated with the RIG-I-like receptor signaling pathway, cytosolic DNA-sensing pathway, Hedgehog signaling pathway, IL-17 signaling pathway, inflammatory bowel disease, and Toll-like receptor signaling pathway. These findings underscore the association of key proteins within the innate immune system with a diverse array of signaling pathways, implying their potential to influence cellular fate through the regulation of various signaling cascades.

### Single-cell transcriptomic analysis of innate immune pathway in cholangiocarcinoma

**Figure 9:**
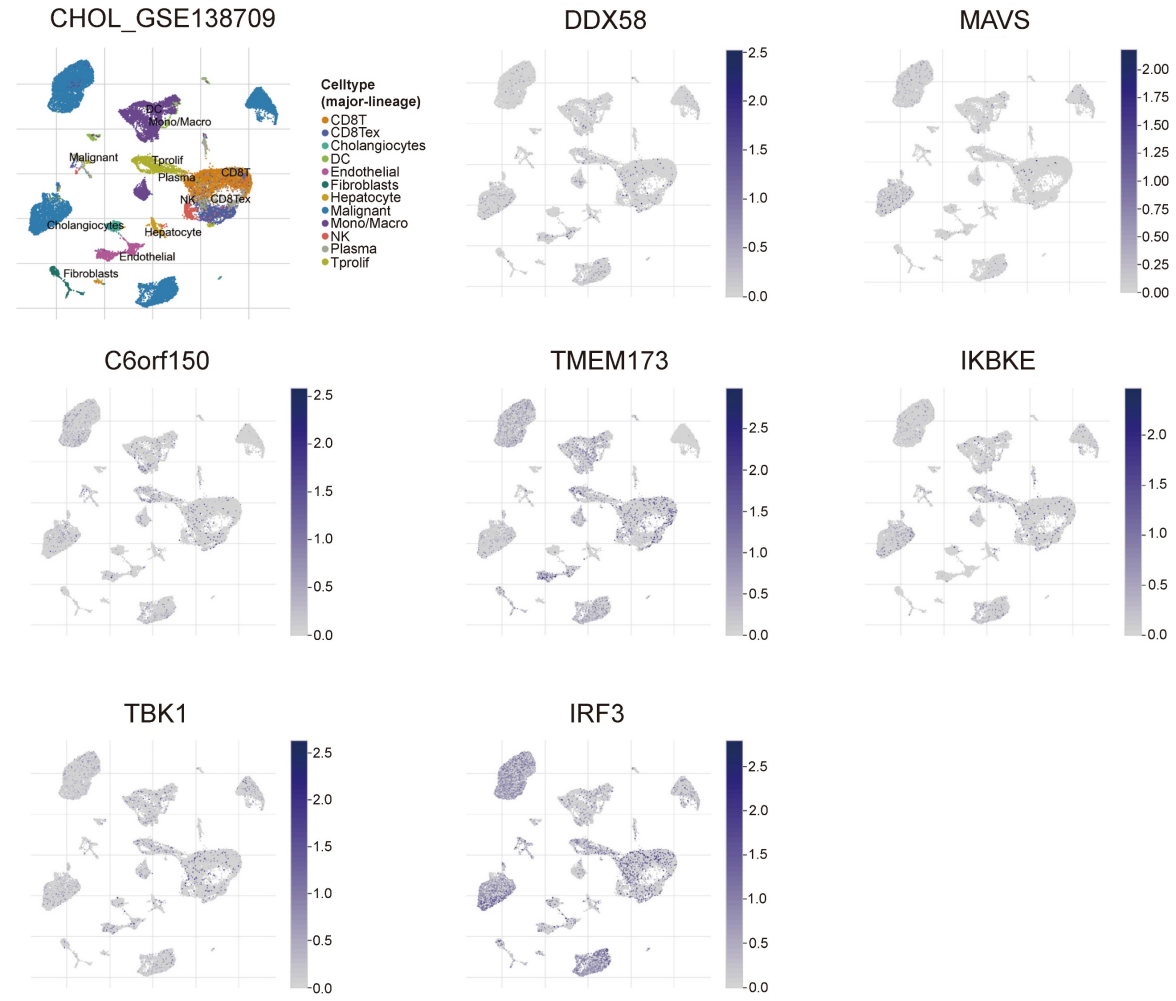
Analysis of differential expression of the innate immune pathway across distinct cellular subtypes in cholangiocarcinoma (single-cell transcriptomic dataset GSE138709). TISCH2 analysis reveals the correlation between innate immune pathway expression and immune cell populations in cholangiocarcinoma.

To gain a deeper understanding of the relevance and underlying mechanisms of the innate immune pathway in cholangiocarcinoma, we investigated the expression levels of *DDX58*, *MAVS*, *C6orf150*, *TMEM173*, *IKBKE*, *TBK1,* and *IRF3* in different cell types using the TISCH2 database. These genes were scrutinized at the single-cell level within 12 cell types, including CD8+ T cells, CD8+ Tex cells, Cholangiocytes, DCs, Endothelial cells, Fibroblasts, Hepatocytes, Malignant cells, monocytes/macrophages, NK cells, plasma and Tprolif cells. In the results of the single-cell transcriptome analysis of cholangiocarcinoma (GSE138709), the aforementioned genes were predominantly expressed in malignant cells, endothelial cells, CD8+ T cells, DCs, and Monocytes/Macrophages (Figure 9). Another single-cell transcriptome dataset for cholangiocarcinoma, GSE142784, revealed their primary expression in endothelial cells and myofibroblasts (Figure S9).

### Immunohistochemical analysis of innate immune pathway in cholangiocarcinoma

**Figure 10:**
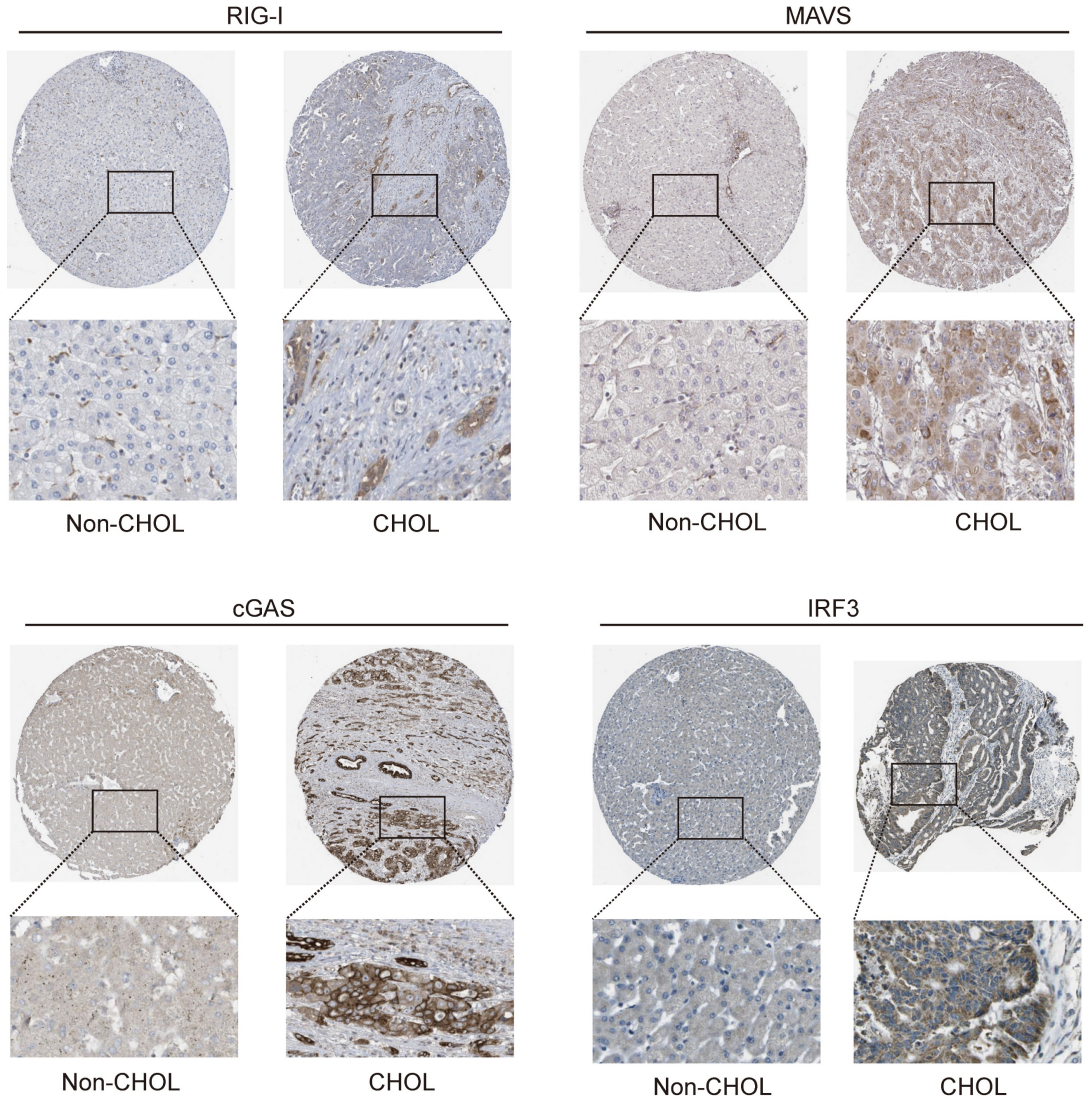
Expression of RIG-I, MAVS, cGAS and IRF3 in normal and intrahepatic cholangiocarcinoma tissues. IHC images demonstrating RIG-I, MAVS, cGAS and IRF3 expression in normal liver tissue (left) or intrahepatic cholangiocarcinoma liver tissue (right).

Tissue samples from the HPA database were analyzed to determine the functions of *DDX58*, *MAVS*, *C6orf150*, *TMEM173*, *IKBKE*, *TBK1,* and *IRF3* in cholangiocarcinoma cells. Additionally, IHC was conducted to assess protein expression in tissue samples. The results indicated a significant increase in RIG-I (encoding gene *DDX58*), MAVS, cGAS (encoding gene *C6orf150*), and IRF3 expression in CHOL tissue samples compared to that in tissue samples from non-CHOL patients (Figure 10).

Subsequently, we examined the expression levels of IFN-I and ISGs in hepatic tissues of patients with intrahepatic cholangiocarcinoma (iCCA) and their normal counterparts. These findings revealed a notable upregulation in the expression of MDA5, IFIT2, and IRF7 in liver tissue sections from patients with intrahepatic cholangiocarcinoma. Conversely, a modest increase in the expression of IFNA1, IFNB1, and IRF1 was observed in liver tissue sections from patients with intrahepatic cholangiocarcinoma (Figure S10). Based on these findings, we observed a significant upregulation in the expression of innate immune pathway proteins and ISGs in hepatic tissue sections from patients with cholangiocarcinoma (iCCA). This alignment with earlier transcriptomic results further substantiates the persistent activation of the innate immune pathway in cholangiocarcinoma.

### Promoter Methylation analysis of innate immune pathway in cholangiocarcinoma

Gene methylation represents the principal form of epigenetic inheritance, and promoter methylation plays a pivotal role in modulating gene expression. Following the preceding analysis, we observed a notable upregulation in the expression of key proteins within the innate immune pathways in cholangiocarcinoma. Therefore, we aimed to further investigate the potential causes of these alterations in protein expression by analyzing the promoter methylation levels of these key proteins.

**Figure 11:**
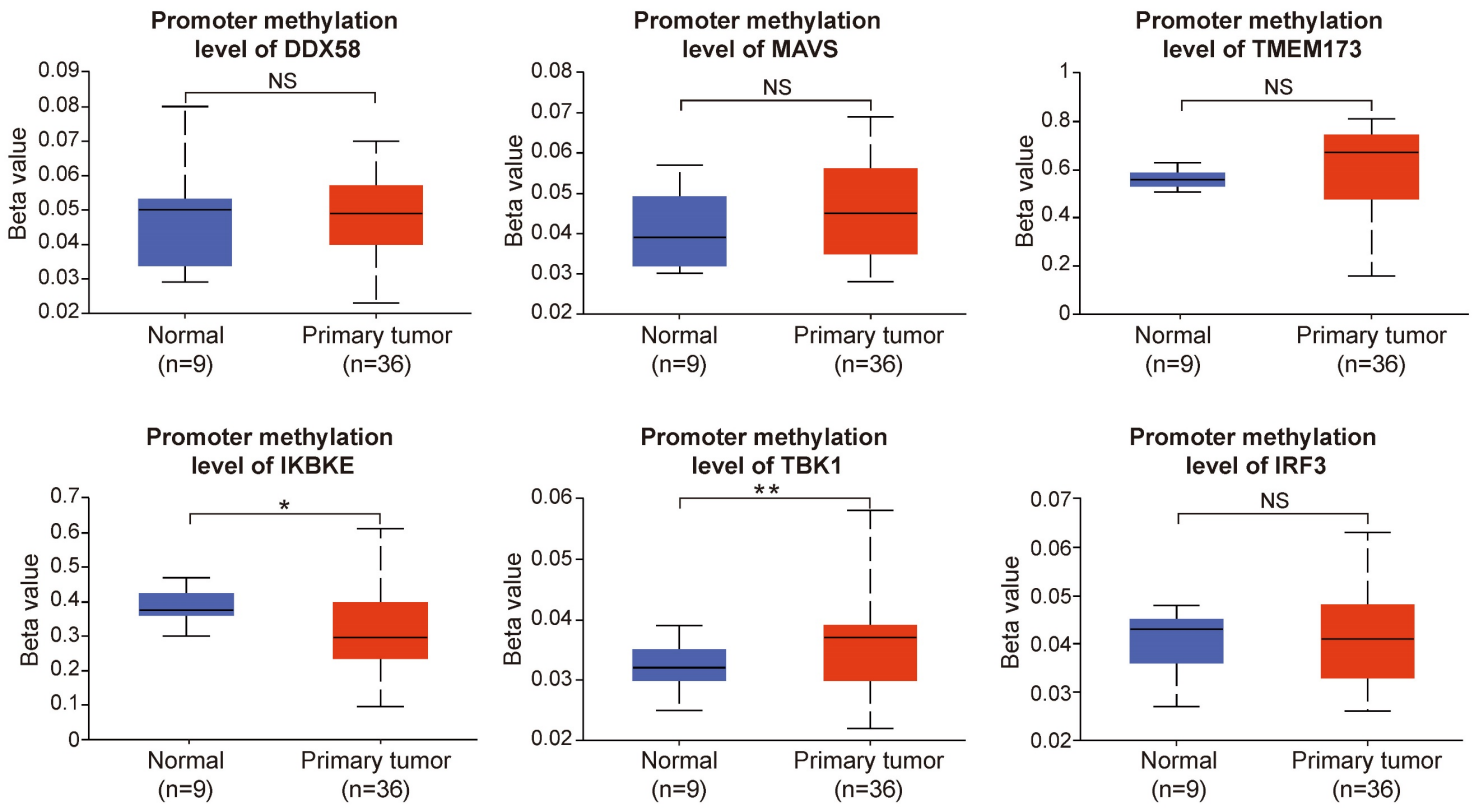
The promoter methylation levels of innate immune pathway in normal and intrahepatic cholangiocarcinoma tissues. The promoter methylation levels of *DDX58*, *MAVS*, *TMEM173*, *IKBKE*, *TBK1*, and *IRF3* in normal and primary tumor tissues (*P < 0.05, **P < 0.01, ***P < 0.001).

Our results revealed that the promoter methylation levels of *DDX58*, *MAVS*, *TMEM173*, and *IRF3* did not exhibit significant differences between normal and tumor samples. However, *IKBKE* exhibited lower methylation levels in tumor samples, whereas *TBK1* showed higher methylation levels in tumor samples compared to normal samples (Figure 11). In summary, the results of promoter methylation for innate immune pathway proteins did not closely align with the expression level results obtained earlier. It is conceivable that additional molecular mechanisms and regulatory factors necessitate further exploration.

### Correlation analysis between the innate immune pathway and intrahepatic metastasis in cholangiocarcinoma

Intrahepatic cholangiocarcinoma (iCCA) is the second most prevalent form of primary liver cancer, constituting approximately 10% -15% of all cases 27. Currently, surgery-based curative resection is the primary modality for iCCA treatment. Nevertheless, 60% of the patients who undergo surgery experience recurrence or metastasis 28, 29. Hence, there is a pressing need to acquire a more profound comprehension of the molecular mechanisms underlying iCCA metastasis and identify novel therapeutic targets to inhibit metastasis, ultimately enhancing the survival outcomes for iCCA patients.

**Figure 12:**
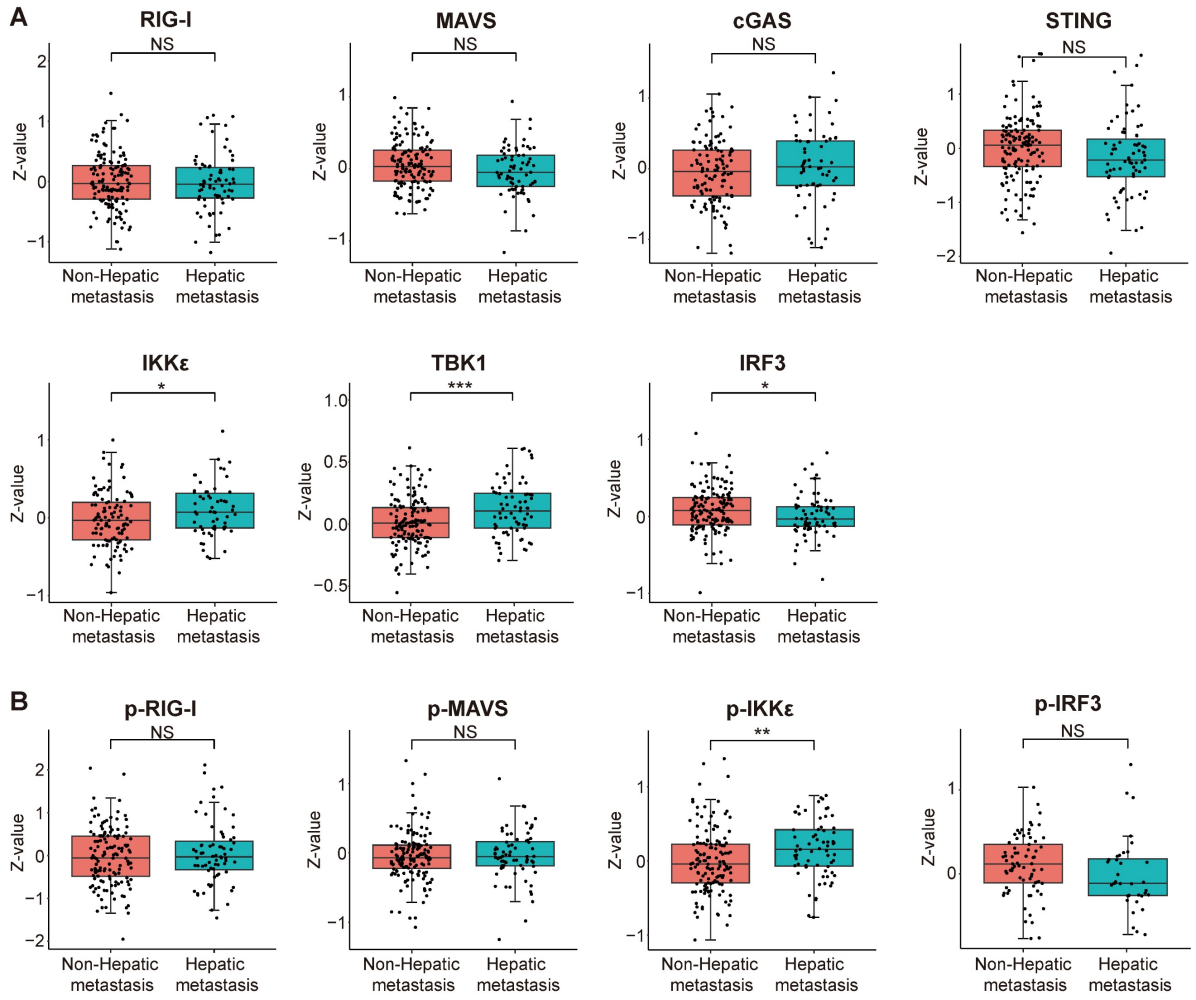
Correlation analysis between the expression and phosphorylation of key proteins within the innate immune pathway and the metastasis of intrahepatic cholangiocarcinoma. (**A**) The relationship between protein expression levels and the metastasis of intrahepatic cholangiocarcinoma. (**B**) The correlation between protein phosphorylation levels and the metastasis of intrahepatic cholangiocarcinoma (*P < 0.05, **P < 0.01, ***P < 0.001).

We scrutinized proteomic data on intrahepatic cholangiocarcinoma published by the Fan team 30. By categorizing proteomic data from 214 intrahepatic cholangiocarcinoma patients based on the presence or absence of intrahepatic metastasis, we investigated disparities in protein expression and variations in phosphorylation intensity of key proteins within the innate immune pathway across different subgroups. The results indicate a significant upregulation of IKKε (encoding gene *IKBKE*) and TBK1 expression but a decrease in IRF3 expression within the intrahepatic cholangiocarcinoma metastasis group. The expression levels of RIG-I, MAVS, cGAS, and STING did not significantly differ between the two groups (Figure 12A). Subsequently, we assessed differences in protein phosphorylation levels between the two groups. The results demonstrate a significant increase in the phosphorylation level of IKKε in the intrahepatic metastasis group. However, the phosphorylation levels of RIG-I, MAVS, and IRF3 did not differ significantly between the two groups (Figure 12B). According to the results, we observed a significant upregulation in both the expression and phosphorylation levels of IKKε protein within the intrahepatic cholangiocarcinoma metastasis group. The protein expression level of TBK1 also exhibited an upward trend in the tumor metastasis group, whereas the expression level of IRF3 demonstrated an opposing phenomenon.

## Discussion

In recent years, an increasing body of research has suggested that innate immune signaling pathways play a crucial role in tumor initiation and progression, as well as in the tumor microenvironment. STING, a pivotal bridging protein involved in antiviral innate immune signaling, is considered a key regulator in antiviral and anti-tumor immunity^31^. Research indicates that, during the early stages of tumor metastasis, elevated expression of the STING pathway in proliferating cancer cells promotes immune system clearance, thereby suppressing the transition from dormant metastasis to malignant recurrence. In contrast, reduced expression of the STING pathway in dormant and malignant metastatic cancer cells facilitates immune evasion^32^. STING functions as a metabolic checkpoint within cells, and its loss in tumor cells increases aerobic glycolysis, promoting lactate accumulation in the tumor microenvironment. This inhibits the immune response to the tumor and accelerates tumor initiation and progression 33. Cui et al. found that STING reshapes the tumor microenvironment by constraining the proliferation of myeloid-derived suppressor cells, thereby suppressing tumor progression and influencing clinical outcomes 34. However, STING signaling activated by STING agonists can induce the generation of regulatory B cells, suppressing the anti-tumor effects of NK cells, thereby hampering the effectiveness of STING agonist immunotherapy in cancer patients 35. In addition, RNA-seq data from a research group led by Charles Spruck indicated activation of antiviral pathways within tumor cells, including RIG-I/MDA5-MAVS, cGAS-STING, interferon IFN pathways, surface antigen presentation pathways, and various cytokines recruiting T cells and NK cells. This activation enhances the immunogenicity of the tumor cells 36. Furthermore, studies have demonstrated that the serine/threonine kinase TBK1 can promote cholangiocarcinoma progression by directly regulating β-catenin phosphorylation 37.

In this study, we examined the expression of key genes involved in the innate immune pathway and their prognostic value across various cancer types, utilizing the TIMER and GEPIA2 databases. Our findings consistently reveal upregulation of innate immune pathway proteins and ISG genes in cholangiocarcinoma compared to normal tissues. Moreover, we observed a consistent increase in ISGs expression associated with the activation of the innate immune pathway in cholangiocarcinoma. This finding further supports the role of the innate immune pathway in cholangiocarcinoma. Subsequently, we conducted an in-depth analysis of alterations in the innate immune pathway in cholangiocarcinoma, using data from TCGA, CPTAC, and GEO databases. This comprehensive analysis encompassed molecular characteristics, such as gene expression, survival prognosis, genetic alterations, and immune infiltration.

Transcriptomic alterations are typically induced by somatic changes in cancer cell genomes. Hence, we analyzed genomic alterations in the innate immune pathway using the cBioPortal database. The results revealed amplification-type alterations in *DDX58*,* TMEM173*, and *IKBKE* in patients with CHOL. Subsequently, we analyzed the relationship between the expression of innate immune pathway proteins and the clinical prognosis of cancer patients. The findings suggested that the expression levels of these pathway proteins were correlated with OS and DFS across diverse tumor types. Notably, in patients with cholangiocarcinoma, the elevated expression of *C6orf150* and *TMEM173* was associated with improved DFS.

Within the tumor microenvironment (TME), both adaptive and innate immune cells infiltrate, thereby regulating tumor progression. Innate immune effectors, encompassing natural killer (NK) cells, eosinophils, basophils, and phagocytic cells, play a pivotal role in tumor suppression through direct cytotoxicity against malignant cells or by eliciting adaptive immune responses. The functionality of the adaptive immune system relies on lymphocytes (B and T cells); B cells primarily contribute to humoral immune responses, whereas T cells participate in cellular immune responses 38-41. Owing to the initiating and regulatory roles of the innate immune pathway in adaptive immune reactions, exploring its correlation with immune cell infiltration in cholangiocarcinoma is crucial. And we observed significant positive correlations between the expression levels of* DDX58*, *C6orf150*, *TMEM173*, *IKBKE*, and *TBK1,* and the infiltration levels of macrophages in cholangiocarcinoma. Interestingly, expression levels of the *C6orf150-TMEM173-IKBKE-TBK1* axis were also significantly and positively correlated with B cell infiltration levels. This suggests that the innate immune pathway in the tumor microenvironment of cholangiocarcinoma not only influences the functionality of innate immune cells but may also be involved in regulating the immune response of adaptive immune cells.

Single-cell sequencing technology can reveal the gene expression status of individual cells, providing in-depth insights into tumor heterogeneity, microenvironment, and tumor cell types. Several studies have conducted single-cell transcriptomic analyses of cholangiocarcinoma, identifying molecular subtypes that differentiate cholangiocarcinoma types and elucidating intercellular communication 42-45. We analyzed two single-cell transcriptome datasets, GSE138709 and GSE142784, and observed that key genes in the innate immune pathway were primarily expressed in malignant cells, endothelial cells, monocytes, and macrophages in cholangiocarcinoma. To further validate the role of the innate immune pathway in cholangiocarcinoma, we analyzed liver tissue slices from patients with CCA and normal control tissues obtained from the HPA database. Immunohistochemical results revealed that the expression of RIG-I, MAVS, cGAS, IRF3, MDA5, IFIT2, and IRF7 was significantly higher in liver tissue slices from patients with cholangiocarcinoma than in the control group. This suggests that the innate immune pathway is continuously activated in the tumor tissues of patients with cholangiocarcinoma.

Most cancer-related deaths are attributed to tumor metastasis 46. Cholangiocarcinoma, an invasive tumor, often prompts patients to seek medical attention in advanced disease stages 47. Effective prevention and treatment of tumor metastasis are thus crucial. We analyzed protein data from patients with intrahepatic cholangiocarcinoma (iCCA) obtained from the research team of Jia Fan 30. Patients with iCCA were divided into two groups based on the intrahepatic metastasis status: metastatic and non-metastatic. The results revealed elevated protein expression and phosphorylation levels of IKKε in the metastatic group, suggesting IKKε as a potential target for treating intrahepatic cholangiocarcinoma metastasis.

The initiation and regulatory roles of innate immune responses in adaptive immune reactions have garnered increasing attention. Furthermore, a growing body of research has explored innate immunity in anti-tumor immune responses and immunotherapy. Moreover, as the second most common malignancy after hepatocellular carcinoma (HCC), cholangiocarcinoma has witnessed a gradual increase in global incidence over the past four decades 48, 49. Hence, investigating the correlation between the activation of the innate immune pathway and cholangiocarcinoma holds potential for providing valuable insights for future therapeutic interventions. Our findings revealed widespread upregulation of interferon-stimulated genes in the tumor tissues of patients with cholangiocarcinoma, suggesting a potential state of chronic activation within the innate immune pathway. This chronic activation may further induce inflammation and exacerbate tumor development and progression, necessitating further investigation into the relationship between the activation of the innate immune pathway and tumors. Despite these findings, several limitations are existing in our current study. The data regarding the innate immune pathway and cholangiocarcinoma samples were retrieved from public databases. Importantly, the reasons behind the consistent upregulation of innate immune pathway protein and ISGs expression in cholangiocarcinoma, particularly the closely associated expression, phosphorylation, and metastasis of IKKε, remain unclear. The detailed mechanisms are yet to be substantiated. The absence of proteomic data on patients with cholangiocarcinoma in public databases precludes the correlation analysis between mRNA and protein expression levels in cholangiocarcinoma tissue samples. Moreover, data pertaining to phosphorylation and other protein modifications of key proteins in the innate immune pathway remain relatively rare within the existing samples of patients with cholangiocarcinoma. Due to the detection of patients with cholangiocarcinoma at advanced stages of cancer progression, the absence of early-stage oncological data precludes the effective correlation between alterations in the innate immune pathway and cancer progression. Hence, further research is warranted to elucidate the intricate molecular mechanisms.

In conclusion, our first pan-cancer analysis of the innate immune pathway showed that the expression of key genes of the innate immune pathway and ISGs in cholangiocarcinoma was higher than that in normal tissues and was statistically correlated with clinical prognosis and immune cell infiltration. Furthermore, the expression and phosphorylation levels of the key kinase IKKε in the innate immune pathway were significantly associated with the metastasis of cholangiocarcinoma. These results contribute novel insights and potential therapeutic targets for cholangiocarcinoma treatment.

## Supplementary Material

Supplementary figures.
