# Supplementary material for: Exploring the role of innate immunity in Cholangiocarcinoma: implications for prognosis, immune infiltration, and tumor metastasis
Source: J Cancer. 2024 May 5;15(11):3547–65. doi: 10.7150/jca.94194 (PMC11134439; doi:10.7150/jca.94194)
Supplement: Supplementary file 1 — Supplementary figures. [file jcav15p3547s1.pdf]

Figure S1, Expression levels of the innate immune pathway across various tumors. For the types of BRCA, CHOL, COAD, ESCA, LIHC, LUSC and STAD in the TCGA project, the corresponding normal tissues in the GTEx database were included as controls. The box plot data were supplied. *P<0.01.

Figure S2, The correlation between differentially expressed innate immune pathway and immune cell infiltration in cholangiocarcinoma. The correlations between the transcriptional levels of DDX58 (A), MAVS (B), IKBKE (C), TBK1 (D) and IRF3 (E) with the infiltration of B cells, CD8+ T cells, CD4+ T cells, macrophages, neutrophils, and dendritic cells in cholangiocarcinoma were shown.

Figure S3, Analysis of differentially expressed genes in correlation with MAVS in cholangiocarcinoma. (A) Volcano plot showing the up-regulated and down-regulated genes correlated with MAVS expression (Pearson test). The significantly positively correlated (B) and negatively correlated (C) genes were shown in heatmaps. (D) Kyoto Encyclopedia Genes and Genomes (KEGG) pathway analysis of the significantly differentially expressed genes in correlation with MAVS. (E) Gene ontology (GO) analysis of the significantly differentially expressed genes in correlation with MAVS.

Figure S4, Analysis of differentially expressed genes in correlation with C6orf150 in cholangiocarcinoma. (A) Volcano plot showing the up-regulated and down-regulated genes correlated with C6orf150 expression (Pearson test). The significantly positively correlated (B) and negatively correlated (C) genes were shown in heatmaps. (D) Kyoto Encyclopedia Genes and Genomes (KEGG) pathway analysis of the significantly differentially expressed genes in correlation with C6orf150. (E) Gene ontology (GO) analysis of the significantly differentially expressed genes in correlation with C6orf150.

Figure S5, Analysis of differentially expressed genes in correlation with TMEM173 in cholangiocarcinoma. (A) Volcano plot showing the up-regulated and down-regulated genes correlated with TMEM173 expression (Pearson test). The significantly positively correlated (B) and

negatively correlated (C) genes were shown in heatmaps. (D) Kyoto Encyclopedia Genes and Genomes (KEGG) pathway analysis of the significantly differentially expressed genes in correlation with TMEM173. (E) Gene ontology (GO) analysis of the significantly differentially expressed genes in correlation with TMEM173.

Figure S6, Analysis of differentially expressed genes in correlation with IKBKE in cholangiocarcinoma. (A) Volcano plot showing the up-regulated and down-regulated genes correlated with IKBKE expression (Pearson test). The significantly positively correlated (B) and negatively correlated (C) genes were shown in heatmaps. (D) Kyoto Encyclopedia Genes and Genomes (KEGG) pathway analysis of the significantly differentially expressed genes in correlation with IKBKE. (E) Gene ontology (GO) analysis of the significantly differentially expressed genes in correlation with IKBKE.

Figure S7, Analysis of differentially expressed genes in correlation with TBK1 in cholangiocarcinoma. (A) Volcano plot showing the up-regulated and down-regulated genes correlated with TBK1 expression (Pearson test). The significantly positively correlated (B) and negatively correlated (C) genes were shown in heatmaps. (D) Kyoto Encyclopedia Genes and Genomes (KEGG) pathway analysis of the significantly differentially expressed genes in correlation with TBK1. (E) Gene ontology (GO) analysis of the significantly differentially expressed genes in correlation with TBK1.

Figure S8, Analysis of differentially expressed genes in correlation with IRF3 in cholangiocarcinoma. (A) Volcano plot showing the up-regulated and down-regulated genes correlated with IRF3 expression (Pearson test). The significantly positively correlated (B) and negatively correlated (C) genes were shown in heatmaps. (D) Kyoto Encyclopedia Genes and Genomes (KEGG) pathway analysis of the significantly differentially expressed genes in correlation with IRF3. (E) Gene ontology (GO) analysis of the significantly differentially expressed genes in correlation with IRF3.

Figure S9, Analysis of differential expression of the antiviral signaling axis across distinct cellular

subtypes in cholangiocarcinoma (single-cell transcriptomic dataset GSE142784). TISCH2 analysis reveals the correlation between antiviral signaling axis expression and immune cell populations in cholangiocarcinoma.

Figure S10, Expression of IFNA1, IFNB1, MDA5, IFIT2, IRF1 and IRF7 in normal and intrahepatic cholangiocarcinoma tissues. IHC images demonstrating IFNA1, IFNB1, MDA5, IFIT2, IRF1 and IRF7 expression in normal liver tissue (left) or intrahepatic cholangiocarcinoma liver tissue (right).

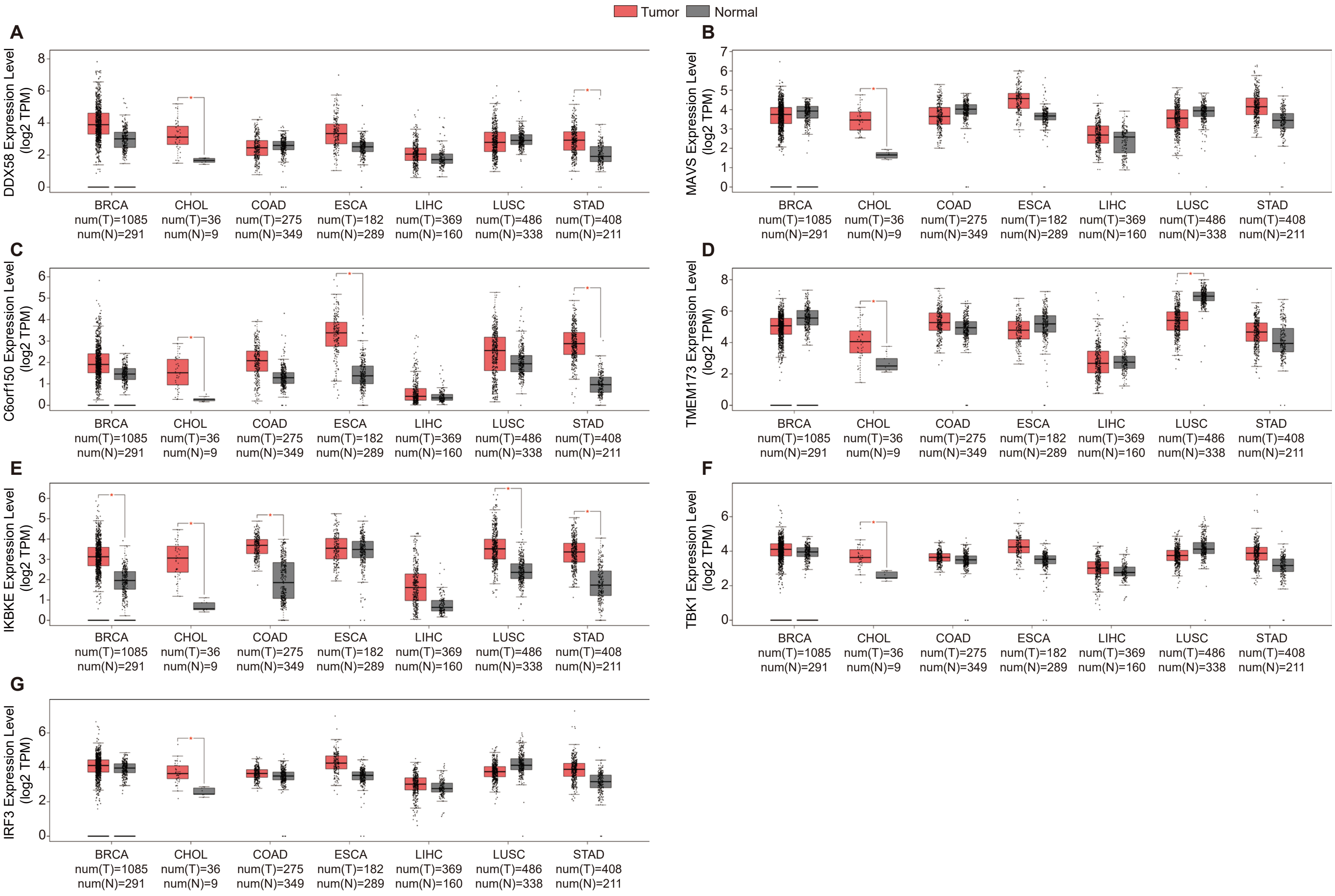
Tumor
Normal
A
DDX58 Expression Level (log2 TPM)
B
MAVS Expression Level (log2 TPM)
C
C6orf150 Expression Level (log2 TPM)
D
TMEM173 Expression Level (log2 TPM)
E
IKBKE Expression Level (log2 TPM)
F
TBK1 Expression Level (log2 TPM)
G
IRF3 Expression Level (log2 TPM)
BRCA num(T)=1085 num(N)=291
CHOL num(T)=36 num(N)=9
COAD num(T)=275 num(N)=349
ESCA num(T)=182 num(N)=289
LIHC num(T)=369 num(N)=160
LUSC num(T)=486 num(N)=338
STAD num(T)=408 num(N)=211

Figure S1

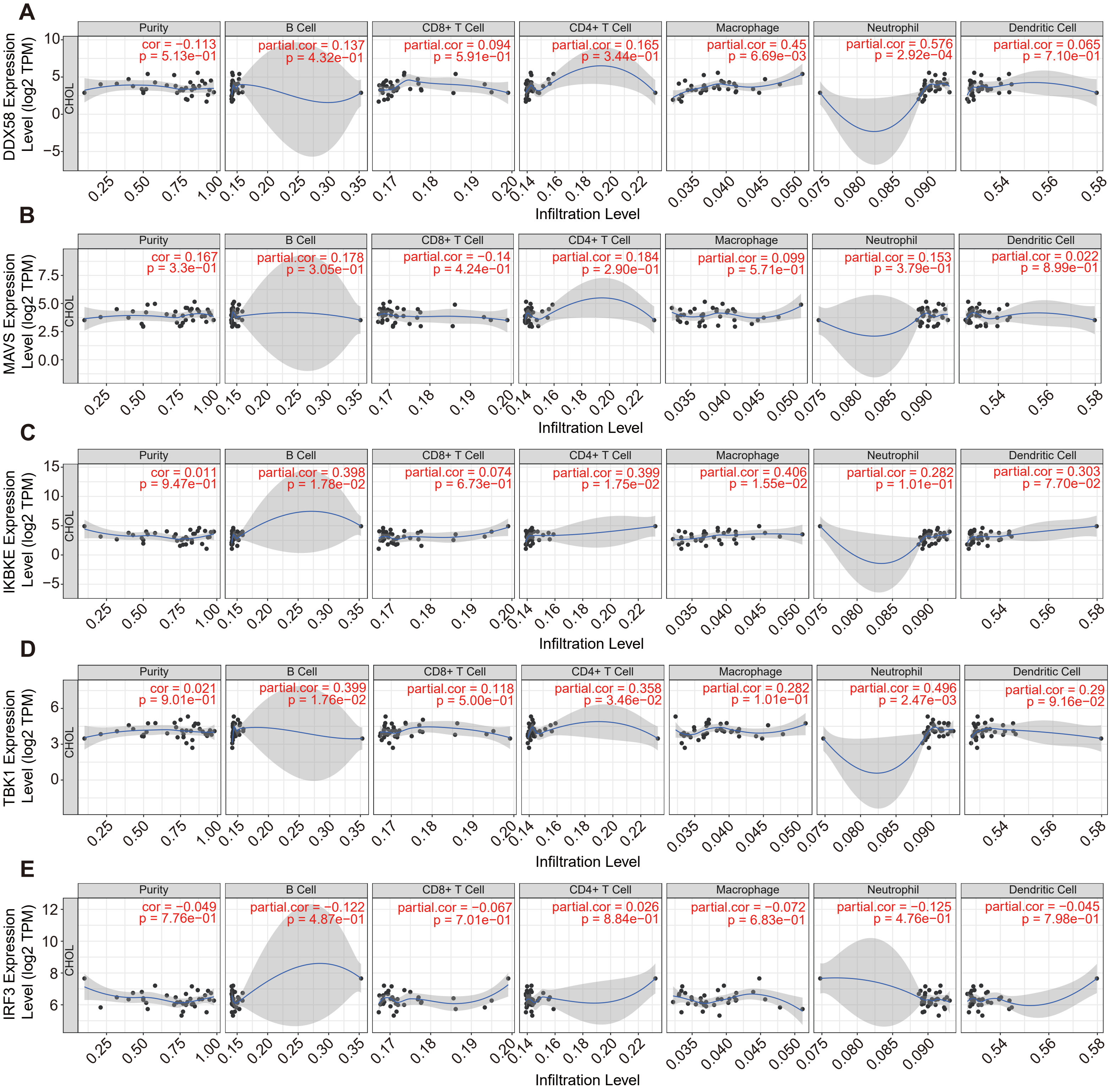
A
DDX58 Expression Level (log2 TPM)
CHOL
Purity
cor = −0.113
p = 5.13e−01
B Cell
partial.cor = 0.137
p = 4.32e−01
CD8+ T Cell
partial.cor = 0.094
p = 5.91e−01
CD4+ T Cell
partial.cor = 0.165
p = 3.44e−01
Macrophage
partial.cor = 0.45
p = 6.69e−03
Neutrophil
partial.cor = 0.576
p = 2.92e−04
Dendritic Cell
partial.cor = 0.065
p = 7.10e−01
Infiltration Level
B
MAVS Expression Level (log2 TPM)
CHOL
Purity
cor = 0.167
p = 3.3e−01
B Cell
partial.cor = 0.178
p = 3.05e−01
CD8+ T Cell
partial.cor = −0.14
p = 4.24e−01
CD4+ T Cell
partial.cor = 0.184
p = 2.90e−01
Macrophage
partial.cor = 0.099
p = 5.71e−01
Neutrophil
partial.cor = 0.153
p = 3.79e−01
Dendritic Cell
partial.cor = 0.022
p = 8.99e−01
Infiltration Level
C
IKBKE Expression Level (log2 TPM)
CHOL
Purity
cor = 0.011
p = 9.47e−01
B Cell
partial.cor = 0.398
p = 1.78e−02
CD8+ T Cell
partial.cor = 0.074
p = 6.73e−01
CD4+ T Cell
partial.cor = 0.399
p = 1.75e−02
Macrophage
partial.cor = 0.406
p = 1.55e−02
Neutrophil
partial.cor = 0.282
p = 1.01e−01
Dendritic Cell
partial.cor = 0.303
p = 7.70e−02
Infiltration Level
D
TBK1 Expression Level (log2 TPM)
CHOL
Purity
cor = 0.021
p = 9.01e−01
B Cell
partial.cor = 0.399
p = 1.76e−02
CD8+ T Cell
partial.cor = 0.118
p = 5.00e−01
CD4+ T Cell
partial.cor = 0.358
p = 3.46e−02
Macrophage
partial.cor = 0.282
p = 1.01e−01
Neutrophil
partial.cor = 0.496
p = 2.47e−03
Dendritic Cell
partial.cor = 0.29
p = 9.16e−02
Infiltration Level
E
IRF3 Expression Level (log2 TPM)
CHOL
Purity
cor = −0.049
p = 7.76e−01
B Cell
partial.cor = −0.122
p = 4.87e−01
CD8+ T Cell
partial.cor = −0.067
p = 7.01e−01
CD4+ T Cell
partial.cor = 0.026
p = 8.84e−01
Macrophage
partial.cor = −0.072
p = 6.83e−01
Neutrophil
partial.cor = −0.125
p = 4.76e−01
Dendritic Cell
partial.cor = −0.045
p = 7.98e−01
Infiltration Level

Figure S2

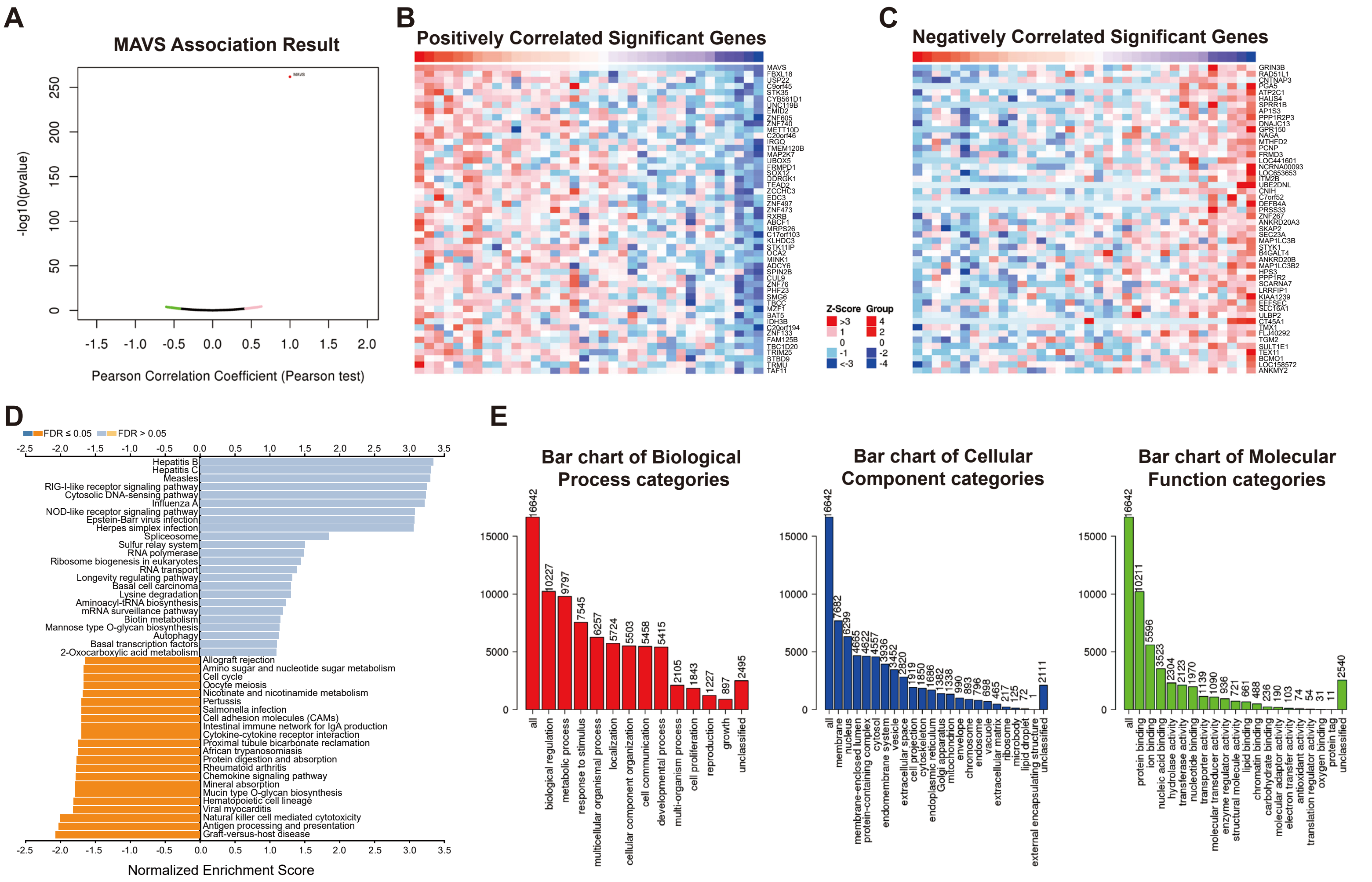
A
MAVS Association Result
-log10(pvalue)
Pearson Correlation Coefficient (Pearson test)
B
Positively Correlated Significant Genes
C
Negatively Correlated Significant Genes
Z-Score
Group
D
FDR ≤ 0.05
FDR > 0.05
Normalized Enrichment Score
E
Bar chart of Biological Process categories
Bar chart of Cellular Component categories
Bar chart of Molecular Function categories

Figure S3

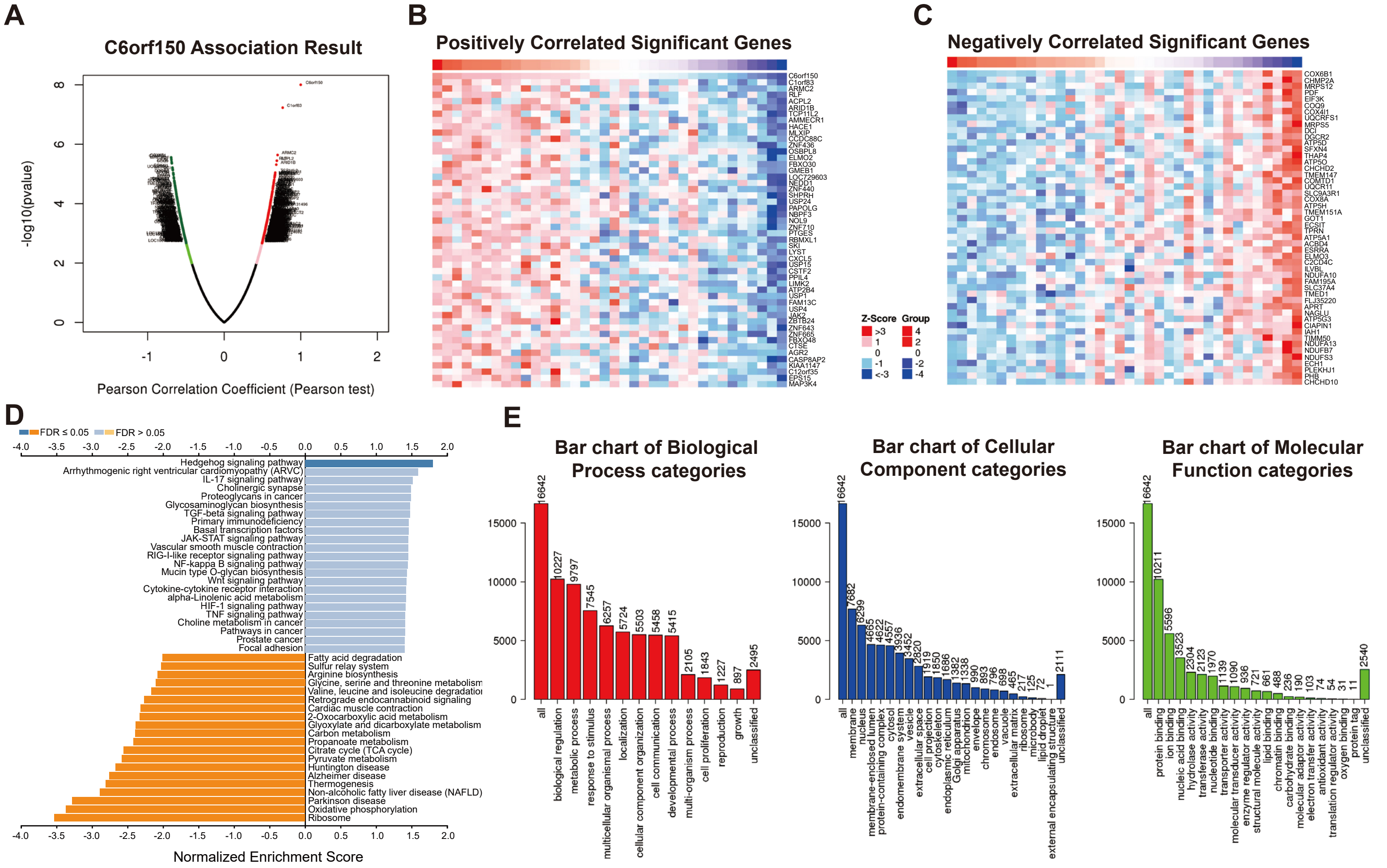
A
C6orf150 Association Result
-log10(pvalue)
Pearson Correlation Coefficient (Pearson test)
B
Positively Correlated Significant Genes
C
Negatively Correlated Significant Genes
Z-Score
Group
D
FDR ≤ 0.05
FDR > 0.05
Hedgehog signaling pathway
Arrhythmogenic right ventricular cardiomyopathy (ARVC)
IL-17 signaling pathway
Cholinergic synapse
Proteoglycans in cancer
Glycosaminoglycan biosynthesis
TGF-beta signaling pathway
Primary immunodeficiency
Basal transcription factors
JAK-STAT signaling pathway
Vascular smooth muscle contraction
RIG-I-like receptor signaling pathway
NF-kappa B signaling pathway
Mucin type O-glycan biosynthesis
Wnt signaling pathway
Cytokine-cytokine receptor interaction
alpha-Linolenic acid metabolism
HIF-1 signaling pathway
TNF signaling pathway
Choline metabolism in cancer
Pathways in cancer
Prostate cancer
Focal adhesion
Fatty acid degradation
Sulfur relay system
Arginine biosynthesis
Glycine, serine and threonine metabolism
Valine, leucine and isoleucine degradation
Retrograde endocannabinoid signaling
Cardiac muscle contraction
2-Oxocarboxylic acid metabolism
Glyoxylate and dicarboxylate metabolism
Carbon metabolism
Propanoate metabolism
Citrate cycle (TCA cycle)
Pyruvate metabolism
Huntington disease
Alzheimer disease
Thermogenesis
Non-alcoholic fatty liver disease (NAFLD)
Parkinson disease
Oxidative phosphorylation
Ribosome
Normalized Enrichment Score
E
Bar chart of Biological Process categories
all 16642
biological regulation 10227
metabolic process 9797
response to stimulus 7545
multicellular organismal process 6257
localization 5724
cellular component organization 5503
cell communication 5458
developmental process 5415
multi-organism process 2105
cell proliferation 1843
reproduction 1227
growth 897
unclassified 2495
Bar chart of Cellular Component categories
all 16642
membrane 7682
nucleus 6299
membrane-enclosed lumen 4665
protein-containing complex 4622
cytosol 4557
endomembrane system 3936
vesicle 3452
extracellular space 2820
cell projection 1919
cytoskeleton 1850
endoplasmic reticulum 1686
Golgi apparatus 1382
mitochondrion 1338
envelope 990
chromosome 893
endosome 796
vacuole 698
extracellular matrix 465
ribosome 217
microbody 125
lipid droplet 72
external encapsulating structure 1
unclassified 2111
Bar chart of Molecular Function categories
all 16642
protein binding 10211
ion binding 5596
nucleic acid binding 3523
hydrolase activity 2304
transferase activity 2123
nucleotide binding 1970
transporter activity 1139
molecular transducer activity 1090
enzyme regulator activity 936
structural molecule activity 721
lipid binding 661
chromatin binding 488
carbohydrate binding 236
molecular adaptor activity 190
electron transfer activity 103
antioxidant activity 74
translation regulator activity 54
oxygen binding 31
protein tag 11
unclassified 2540

Figure S4

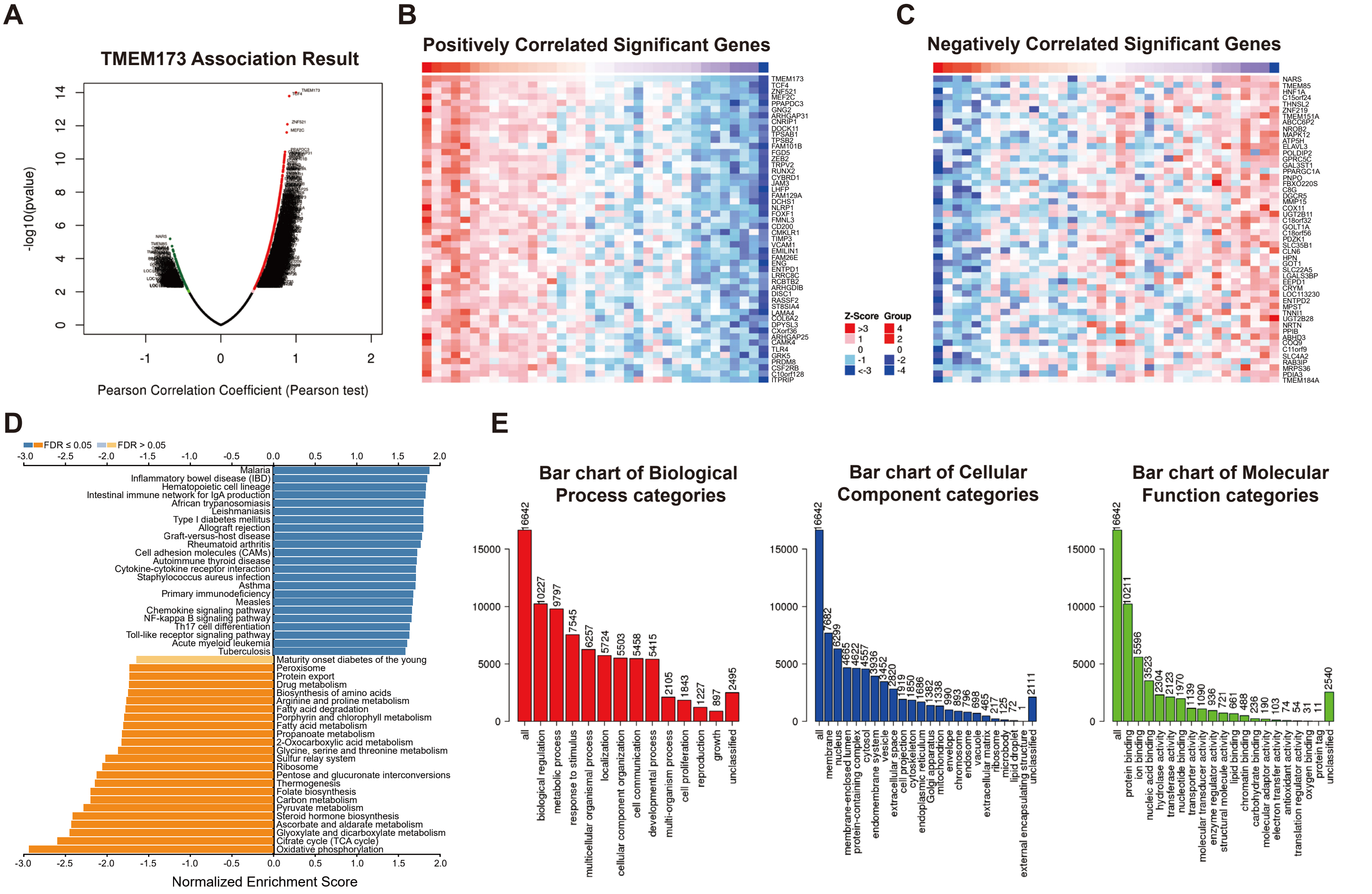
A
TMEM173 Association Result
-log10(pvalue)
Pearson Correlation Coefficient (Pearson test)
B
Positively Correlated Significant Genes
C
Negatively Correlated Significant Genes
Z-Score
Group
D
FDR ≤ 0.05
FDR > 0.05
Malaria
Inflammatory bowel disease (IBD)
Hematopoietic cell lineage
Intestinal immune network for IgA production
African trypanosomiasis
Leishmaniasis
Type I diabetes mellitus
Allograft rejection
Graft-versus-host disease
Rheumatoid arthritis
Cell adhesion molecules (CAMs)
Autoimmune thyroid disease
Cytokine-cytokine receptor interaction
Staphylococcus aureus infection
Asthma
Primary immunodeficiency
Measles
Chemokine signaling pathway
NF-kappa B signaling pathway
Th17 cell differentiation
Toll-like receptor signaling pathway
Acute myeloid leukemia
Tuberculosis
Maturity onset diabetes of the young
Peroxisome
Protein export
Drug metabolism
Biosynthesis of amino acids
Arginine and proline metabolism
Fatty acid degradation
Porphyrin and chlorophyll metabolism
Fatty acid metabolism
Propanoate metabolism
2-Oxocarboxylic acid metabolism
Glycine, serine and threonine metabolism
Sulfur relay system
Ribosome
Pentose and glucuronate interconversions
Thermogenesis
Folate biosynthesis
Carbon metabolism
Pyruvate metabolism
Steroid hormone biosynthesis
Ascorbate and aldarate metabolism
Glyoxylate and dicarboxylate metabolism
Citrate cycle (TCA cycle)
Oxidative phosphorylation
Normalized Enrichment Score
E
Bar chart of Biological Process categories
Bar chart of Cellular Component categories
Bar chart of Molecular Function categories

Figure S5

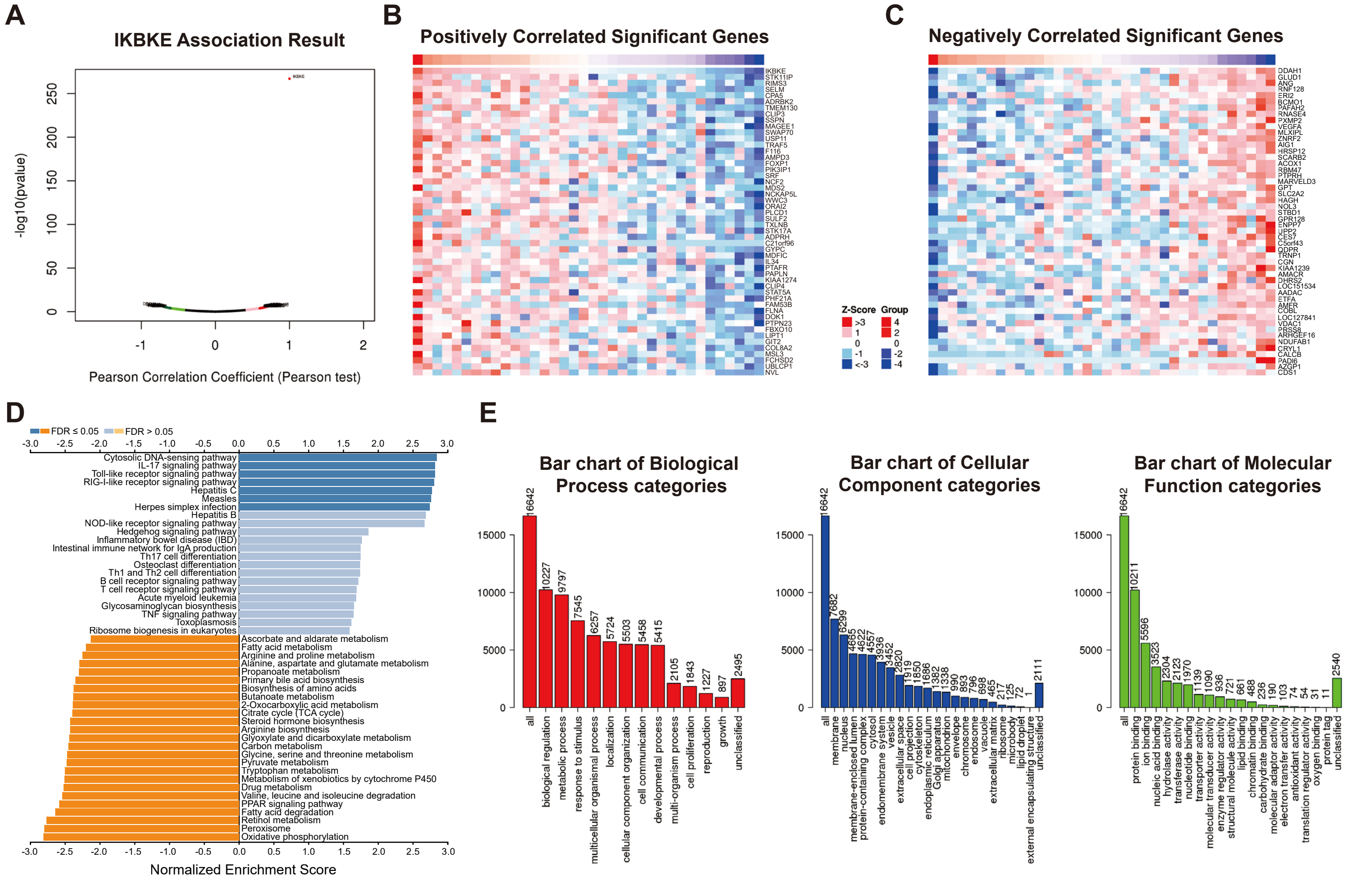
A
IKBKE Association Result
-log10(pvalue)
Pearson Correlation Coefficient (Pearson test)
B
Positively Correlated Significant Genes
C
Negatively Correlated Significant Genes
Z-Score
Group
D
FDR ≤ 0.05
FDR > 0.05
Cytosolic DNA-sensing pathway
IL-17 signaling pathway
Toll-like receptor signaling pathway
RIG-I-like receptor signaling pathway
Hepatitis C
Measles
Herpes simplex infection
Hepatitis B
NOD-like receptor signaling pathway
Hedgehog signaling pathway
Inflammatory bowel disease (IBD)
Intestinal immune network for IgA production
Th17 cell differentiation
Osteoclast differentiation
Th1 and Th2 cell differentiation
B cell receptor signaling pathway
T cell receptor signaling pathway
Acute myeloid leukemia
Glycosaminoglycan biosynthesis
TNF signaling pathway
Toxoplasmosis
Ribosome biogenesis in eukaryotes
Ascorbate and aldarate metabolism
Fatty acid metabolism
Arginine and proline metabolism
Alanine, aspartate and glutamate metabolism
Propanoate metabolism
Primary bile acid biosynthesis
Biosynthesis of amino acids
Butanoate metabolism
2-Oxocarboxylic acid metabolism
Citrate cycle (TCA cycle)
Steroid hormone biosynthesis
Arginine biosynthesis
Glyoxylate and dicarboxylate metabolism
Carbon metabolism
Glycine, serine and threonine metabolism
Pyruvate metabolism
Tryptophan metabolism
Metabolism of xenobiotics by cytochrome P450
Drug metabolism
Valine, leucine and isoleucine degradation
PPAR signaling pathway
Fatty acid degradation
Retinol metabolism
Peroxisome
Oxidative phosphorylation
Normalized Enrichment Score
E
Bar chart of Biological Process categories
all
biological regulation
metabolic process
response to stimulus
multicellular organismal process
localization
cellular component organization
cell communication
developmental process
multi-organism process
cell proliferation
reproduction
growth
unclassified
Bar chart of Cellular Component categories
all
membrane
nucleus
membrane-enclosed lumen
protein-containing complex
cytosol
endomembrane system
vesicle
extracellular space
cell projection
cytoskeleton
endoplasmic reticulum
Golgi apparatus
mitochondrion
envelope
chromosome
endosome
vacuole
extracellular matrix
ribosome
microbody
lipid droplet
external encapsulating structure
unclassified
Bar chart of Molecular Function categories
all
protein binding
ion binding
nucleic acid binding
hydrolase activity
transferase activity
nucleotide binding
transporter activity
molecular transducer activity
enzyme regulator activity
structural molecule activity
lipid binding
chromatin binding
carbohydrate binding
molecular adaptor activity
electron transfer activity
antioxidant activity
translation regulator activity
oxygen binding
protein tag
unclassified

Figure S6

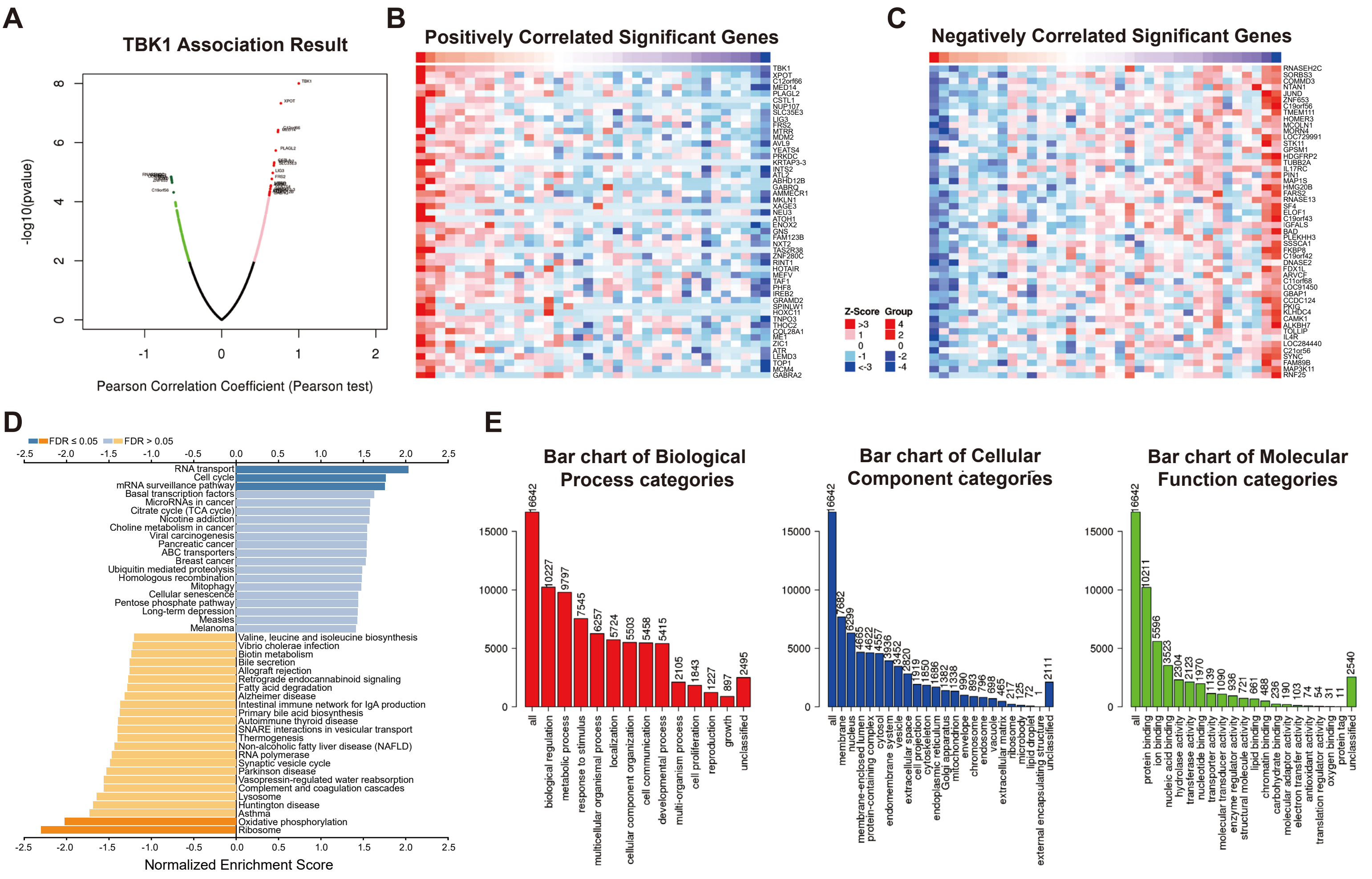
A
TBK1 Association Result
-log10(pvalue)
Pearson Correlation Coefficient (Pearson test)
B
Positively Correlated Significant Genes
C
Negatively Correlated Significant Genes
Z-Score
Group
D
FDR ≤ 0.05
FDR > 0.05
Normalized Enrichment Score
RNA transport
Cell cycle
mRNA surveillance pathway
Basal transcription factors
MicroRNAs in cancer
Citrate cycle (TCA cycle)
Nicotine addiction
Choline metabolism in cancer
Viral carcinogenesis
Pancreatic cancer
ABC transporters
Breast cancer
Ubiquitin mediated proteolysis
Homologous recombination
Mitophagy
Cellular senescence
Pentose phosphate pathway
Long-term depression
Measles
Melanoma
Valine, leucine and isoleucine biosynthesis
Vibrio cholerae infection
Biotin metabolism
Bile secretion
Allograft rejection
Retrograde endocannabinoid signaling
Fatty acid degradation
Alzheimer disease
Intestinal immune network for IgA production
Primary bile acid biosynthesis
Autoimmune thyroid disease
SNARE interactions in vesicular transport
Thermogenesis
Non-alcoholic fatty liver disease (NAFLD)
RNA polymerase
Synaptic vesicle cycle
Parkinson disease
Vasopressin-regulated water reabsorption
Complement and coagulation cascades
Lysosome
Huntington disease
Asthma
Oxidative phosphorylation
Ribosome
E
Bar chart of Biological Process categories
Bar chart of Cellular Component categories
Bar chart of Molecular Function categories

Figure S7

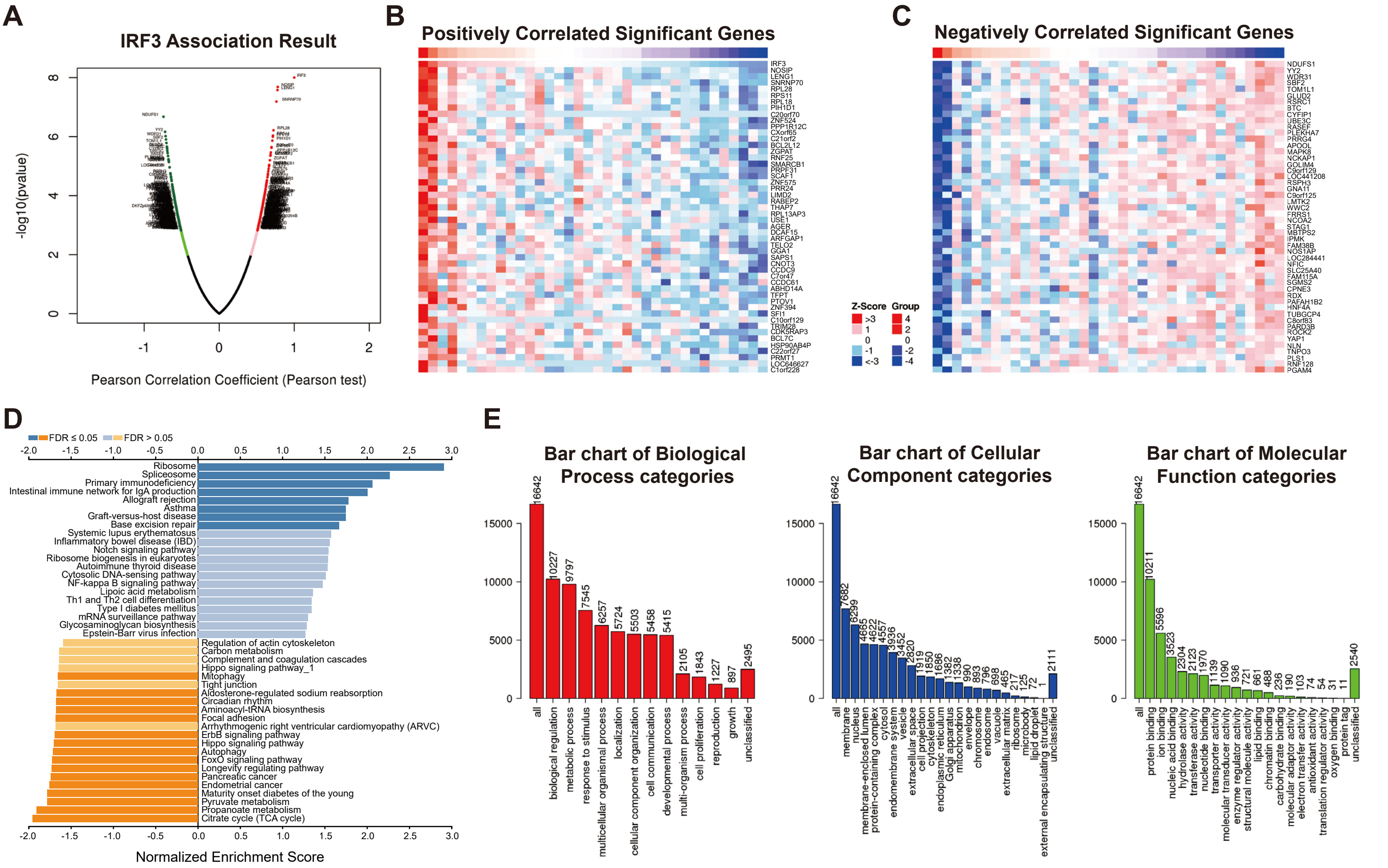
A
IRF3 Association Result
-log10(pvalue)
Pearson Correlation Coefficient (Pearson test)
B
Positively Correlated Significant Genes
C
Negatively Correlated Significant Genes
Z-Score
Group
D
FDR ≤ 0.05
FDR > 0.05
Ribosome
Spliceosome
Primary immunodeficiency
Intestinal immune network for IgA production
Allograft rejection
Asthma
Graft-versus-host disease
Base excision repair
Systemic lupus erythematosus
Inflammatory bowel disease (IBD)
Notch signaling pathway
Ribosome biogenesis in eukaryotes
Autoimmune thyroid disease
Cytosolic DNA-sensing pathway
NF-kappa B signaling pathway
Lipoic acid metabolism
Th1 and Th2 cell differentiation
Type I diabetes mellitus
mRNA surveillance pathway
Glycosaminoglycan biosynthesis
Epstein-Barr virus infection
Regulation of actin cytoskeleton
Carbon metabolism
Complement and coagulation cascades
Hippo signaling pathway_1
Mitophagy
Tight junction
Aldosterone-regulated sodium reabsorption
Circadian rhythm
Aminoacyl-tRNA biosynthesis
Focal adhesion
Arrhythmogenic right ventricular cardiomyopathy (ARVC)
ErbB signaling pathway
Hippo signaling pathway
Autophagy
FoxO signaling pathway
Longevity regulating pathway
Pancreatic cancer
Endometrial cancer
Maturity onset diabetes of the young
Pyruvate metabolism
Propanoate metabolism
Citrate cycle (TCA cycle)
Normalized Enrichment Score
E
Bar chart of Biological Process categories
all
biological regulation
metabolic process
response to stimulus
multicellular organismal process
localization
cellular component organization
cell communication
developmental process
multi-organism process
cell proliferation
reproduction
growth
unclassified
Bar chart of Cellular Component categories
all
membrane
nucleus
membrane-enclosed lumen
protein-containing complex
cytosol
endomembrane system
vesicle
extracellular space
cell projection
cytoskeleton
endoplasmic reticulum
Golgi apparatus
mitochondrion
envelope
chromosome
endosome
vacuole
extracellular matrix
ribosome
microbody
lipid droplet
external encapsulating structure
unclassified
Bar chart of Molecular Function categories
all
protein binding
ion binding
nucleic acid binding
hydrolase activity
transferase activity
nucleotide binding
transporter activity
molecular transducer activity
enzyme regulator activity
structural molecule activity
lipid binding
chromatin binding
carbohydrate binding
molecular adaptor activity
electron transfer activity
antioxidant activity
translation regulator activity
oxygen binding
protein tag
unclassified

Figure S8

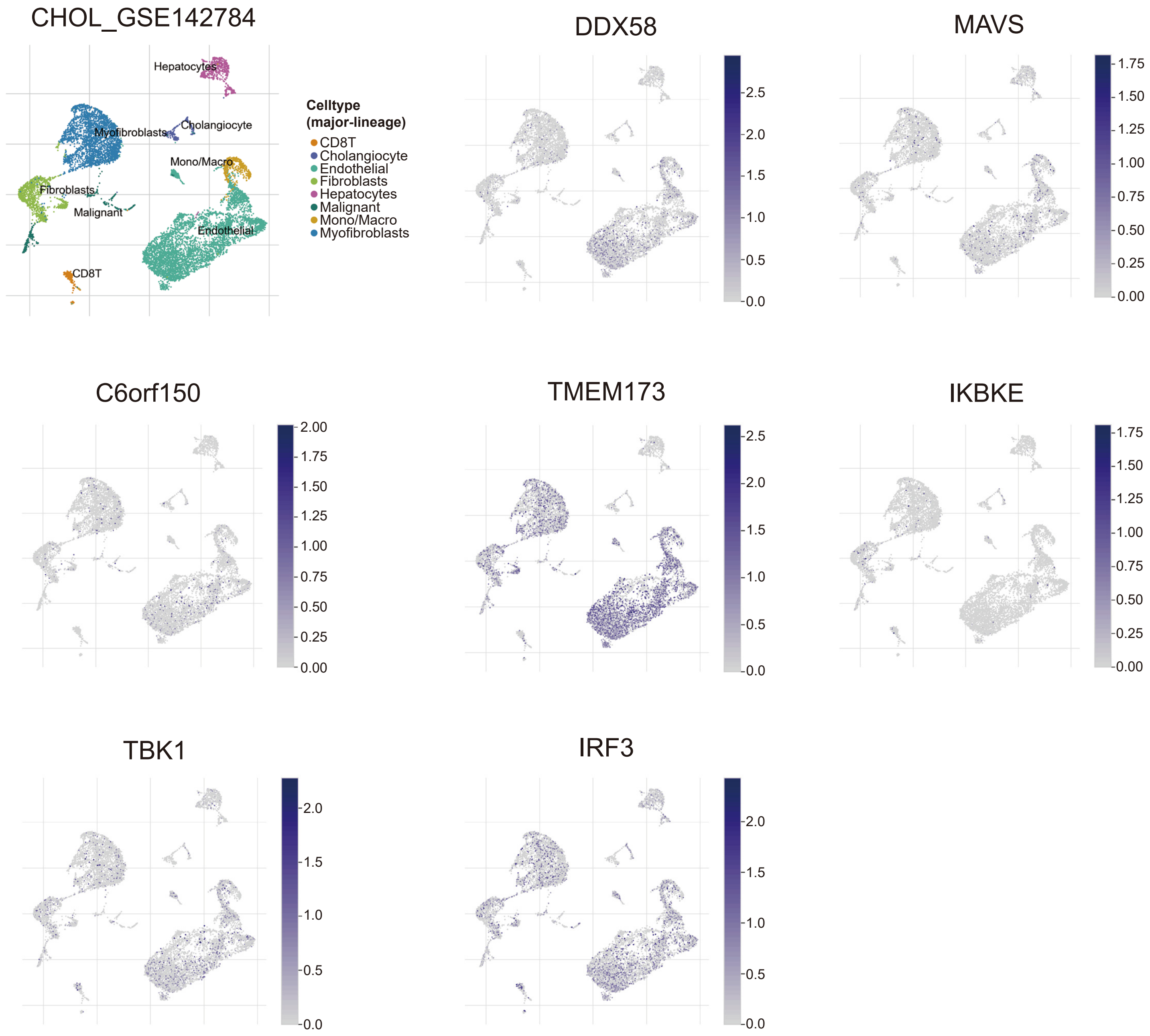
CHOL_GSE142784
Hepatocytes
Myofibroblasts
Cholangiocyte
Mono/Macro
Fibroblasts
Malignant
Endothelial
CD8T
Celltype (major-lineage)
CD8T
Cholangiocyte
Endothelial
Fibroblasts
Hepatocytes
Malignant
Mono/Macro
Myofibroblasts
DDX58
MAVS
C6orf150
TMEM173
IKBKE
TBK1
IRF3

Figure S9

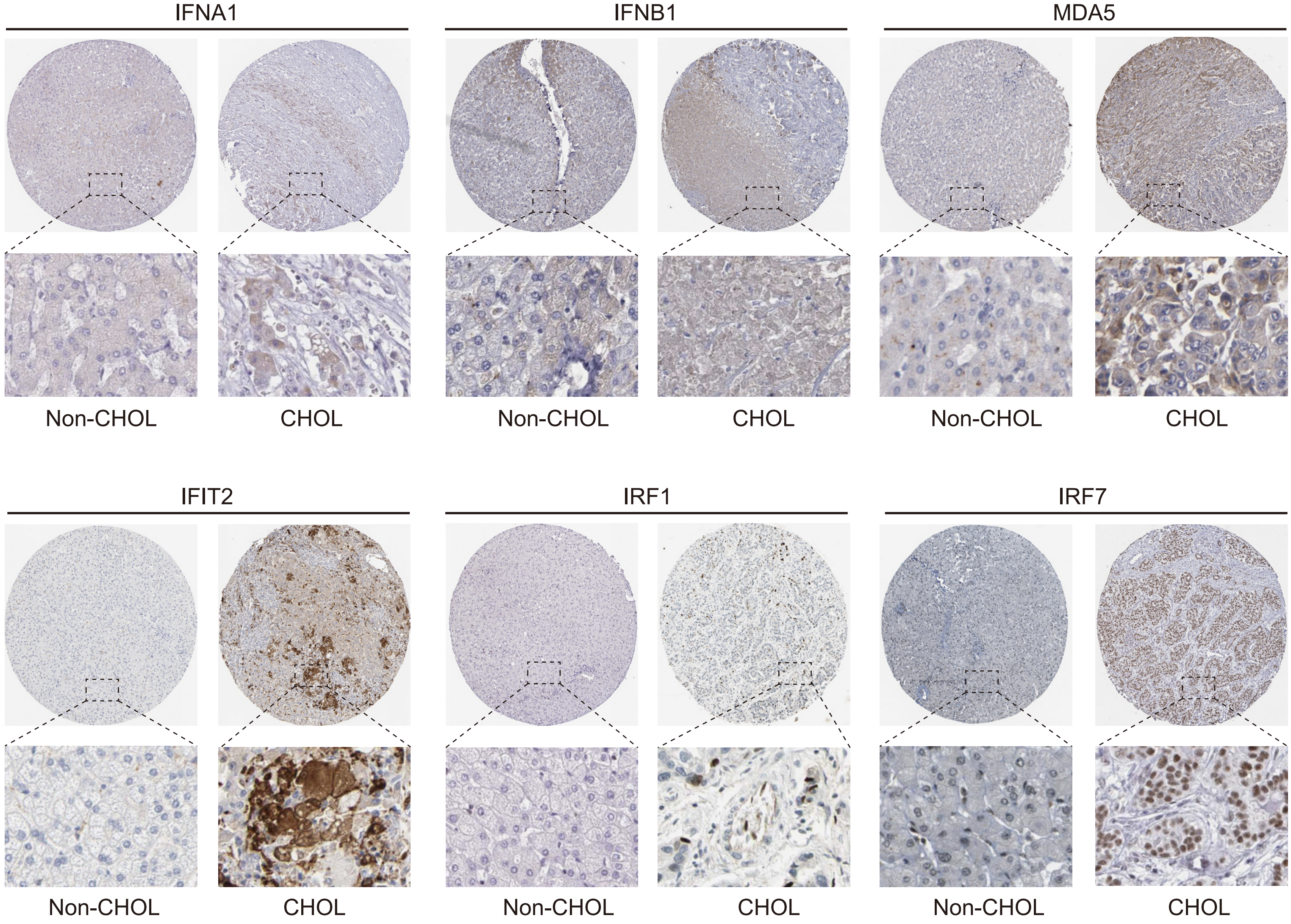
IFNA1
IFNB1
MDA5
Non-CHOL
CHOL
Non-CHOL
CHOL
Non-CHOL
CHOL
IFIT2
IRF1
IRF7
Non-CHOL
CHOL
Non-CHOL
CHOL
Non-CHOL
CHOL

Figure S10
